# The role of pathologists in the diagnosis of occupational lung diseases: an expert opinion of the European Society of Pathology Pulmonary Pathology Working Group

**DOI:** 10.1007/s00428-024-03845-1

**Published:** 2024-07-20

**Authors:** Fiorella Calabrese, M. Angeles Montero-Fernandez, Izidor Kern, Federica Pezzuto, Francesca Lunardi, Paul Hofman, Sabina Berezowska, Richard Attanoos, Louise Burke, Paola Mason, Elisabetta Balestro, Maria Molina Molina, Chiara Giraudo, Helmut Prosch, Luka Brcic, Francoise Galateau-Salle

**Affiliations:** 1 Department of Cardiac, Thoracic, Vascular Sciences, and Public Health, University of Padova, Padova, Italy; 2 Department of Cellular Pathology, Liverpool University Foundation Trust, Liverpool, UK; 3 Cytology and Pathology Laboratory, University Clinic of Respiratory and Allergic Diseases Golnik, Golnik, Slovenia; 4 Laboratory of Clinical and Experimental Pathology, IHU RespirERA, Nice Hospital, University Côte d’Azur, Nice, France; 5 Institute of Pathology, Lausanne University Hospital and University of Lausanne, Lausanne, Switzerland; 6 Department of Cellular Pathology, Cardiff University, Cardiff, UK; 7 Department of Histopathology, Cork University Hospital, Cork, Ireland; 8 University Hospital of Padova, Padua, Italy; 9 Respiratory Department, University Hospital of Bellvitge, IDIBELL, CIBERES, L’Hospitalet de Llobregat, Spain; 10 Division of Radiology, Department of Biomedical Imaging and Image-Guided Therapy, Medical University of Vienna, Vienna, Austria; 11 Diagnostic and Research Centre for Molecular BioMedicine, Diagnostic & Research Institute of Pathology, Medical University of Graz, Graz, Austria; 12 Centre Léon Bérard, MESOPATH College, Lyon, France

**Keywords:** Occupational lung disease, Thoracic diseases, Environmental, Exposure

## Abstract

Occupational lung/thoracic diseases are a major global public health issue. They comprise a diverse spectrum of health conditions with complex pathology, most of which arise following chronic heavy workplace exposures to various mineral dusts, metal fumes, or following inhaled organic particulate reactions. Many occupational lung diseases could become irreversible; thus accurate diagnosis is mandatory to minimize dust exposure and consequently reduce damage to the respiratory system. Lung biopsy is usually required when exposure history is inconsistent with imaging, in case of unusual or new exposures, in case of unexpected malignancy, and in cases in which there are claims for personal injury and legal compensation. In this paper, we provide an overview of the most frequent occupational lung diseases with a focus on pathological diagnosis. This is a paper that summarizes the expert opinion from a group of European pathologists, together with contributions from other specialists who are crucial for the diagnosis and management of these diseases. Indeed, tight collaboration of all specialists involved in the workup is mandatory as many occupational lung diseases are misdiagnosed or go unrecognized. This document provides a guide for pathologists in practice to facilitate the accurate diagnosis of occupational lung disease. The review article reports relevant topics discussed during an educational course held by expert pathologists, active members of the Pulmonary Pathology Working Group of the European Society of Pathology. The course was endorsed by the University of Padova as a “winter school” (selected project in the call for “Shaping a World-class University” 2022).

## Introduction

Occupational lung diseases may follow acute, subacute, or chronic exposure to harmful substances in specific work environments, leading to lasting lung damage even after the exposure ends. They encompass a broad range of both benign and malignant pulmonary conditions that can affect various parts of the respiratory system, from the upper airways to the alveoli, including obstructive diseases like chronic obstructive lung disease (COPD) and asthma, restrictive disorders such as pulmonary fibrosis (PF), mixed obstructive and restrictive lung diseases (e.g., hypersensitivity pneumonitis), and malignancies like mesothelioma and lung cancer [1]. Recent data from the Health and Safety Executive (HSE) discloses that 12,000 fatalities each year are linked to prior workplace exposure-related lung diseases (https://press.hse.gov.uk/2023/06/05/hse-inspections-target-woodworking-businesses-to-tackle-occupational-lung-disease-2/). Approximately 19,000 new instances of lung or respiratory pathology, suspected to be induced or exacerbated by occupational conditions, are reported per year (https://press.hse.gov.uk/2023/06/05/hse-inspections-target-woodworking-businesses-to-tackle-occupational-lung-disease-2/). The precise count of cases with this diagnosis may differ considerably from known statistics, as many instances continue to be characterized as “highly suspicious” or “suggestive” of an occupational disease without conclusive evidence. The presence of ambiguous cases underscores the limitations of current diagnostic tools and the intricacies of occupational health dynamics. These cases often pose a challenge to clinicians, as they display clinical features or occupational exposures suggestive of a potential link to specific diseases but lack definitive evidence to establish causality. Addressing these uncertainties demands a multifaceted approach that integrates clinical expertise, thorough occupational histories, and advanced investigative techniques. Furthermore, it requires acknowledgment of the evolving nature of occupational health research and the necessity for ongoing surveillance to detect emerging occupational hazards and associated health risks.

In industrial settings, the evolution of work environments is a key factor contributing to the rise of occupational thoracic lung diseases. The introduction of new materials and technologies has changed workplaces, potentially exposing workers to different and continuously changing hazardous substances. Globalization further complicates this issue as industries operating on a global scale adhere to varying occupational health and safety standards, risking an increase in workers’ exposure levels. Additionally, informal labour practices prevalent in many countries exacerbate the problem, as the informal sector often lacks stringent occupational safety regulations, which may endanger workers’ respiratory health [2]. Several factors make diagnosing occupational thoracic lung diseases challenging. A significant obstacle is the latency period associated with these diseases, where symptoms may appear a long time after exposure, making it difficult to link the disease to workplace hazards. Symptoms exhibited by affected individuals are often nonspecific, such as cough or shortness of breath, mimicking other respiratory conditions and complicating the diagnosis. Underreporting is another issue, as workers may avoid reporting symptoms or exposure due to job security concerns and lack of awareness about hazards and adequate protections, hindering accurate diagnosis and intervention. Limited access to healthcare services further complicates matters, as some workers may struggle to obtain timely medical attention, thus delaying a correct diagnosis and treatment of occupational lung diseases.

It is well recognized that workers experience heterogeneous exposures, which impact upon the occurrence and severity of disease. There may be an interplay with personal factors determined by genetics such as immune response reactions, and there can be variations in the occurrence and severity of these diseases. This diversity underscores the role of unique host factors that make individuals susceptible to workplace-related respiratory diseases. These factors include genetics, pre-existing health conditions, lifestyle choices, and the duration and intensity of exposure. Moreover, workers often face multiple simultaneous exposures from the most frequent tobacco source to other pollutants, making it difficult to identify the specific cause of the disease and hampering a precise diagnosis and preventive measures [3, 4]. To tackle all these challenges, increased awareness, and close collaboration among specialists from different fields are crucial for preventing, controlling, accurately diagnosing, and managing occupational lung diseases, ultimately minimizing their impact on individuals and the economy. This review, developed after a 1-week “winter school” on occupational lung diseases, provides a detailed pathological overview of the most impacting diseases, with the crucial contribution of the various specialists who should routinely be involved in the complex journey of these patients.

## Sources and tools for pathological diagnosis

Pathological diagnosis related to occupational diseases is very complex. Since many agents are present both in the general environment and in certain professions, the distinction and accurate identification of causative agents may be particularly difficult. For an adequate sampling, clinical and radiological information, and detailed biological or physical attributes of suspected agents are important. For example, inhaled spherical dust particles of 1–5 μm in diameter that have a density similar to water deposit in alveoli. Larger and/or denser particles remain on airway walls and are cleared by ciliary defence mechanisms, while smaller particles are exhaled. Upper lobes accumulate more dust particles, except for asbestos fibres, showing a length:diameter ratio of 3:1. In a standing position at rest, the upper parts of the lung have poor perfusion and aeration leading to an inability to eliminate encountered dusts [5, 6]. Different sample types, such as bronchoalveolar lavage (BAL), sputum, and lung biopsies or resection may be used for diagnosis [7]. BAL, when combined with histology and specialized techniques, can be particularly useful [7], as the informative role of BAL in hard metal pneumoconiosis, sometimes in asbestosis, silicosis/silicatosis, and in hypersensitivity pneumonitis (HP) after exposure to organic agents [7]. Besides the detection of mineral particles, to a certain extent supported by special stains, the identification of a typical inflammatory background may be useful not only in the diagnosis of disease such as organic agent pulmonary diseases (HP) but also in better understanding the complex pathogenetic mechanisms of these entities [8]. Furthermore, the use of these minimally invasive procedures (BAL or transbronchial biopsy) is undeniably adequate to exclude mimickers that may cause clinical features of alveolitis [8]. The more recent implementation of the use of cryoprobes has allowed us to achieve transbronchial sampling of 1–3 cm of lung (i.e., cryobiopsy) with better-preserved tissue architecture and a higher diagnostic yield with a lesser mortality/morbidity [8]. Regarding the video-thoracoscopic surgical lung biopsies, several factors need consideration—the lung region to be biopsied (upper or lower lobe based on the suspected agent), the location of sampling (typically increased subpleural fibre accumulation), and strategies to mitigate the challenges due to heterogeneity. As for other diffuse lung diseases, the surgical lung biopsy should be of adequate size (measuring 3 to 5 cm in length and 3 cm in depth) avoiding the tips of the lobes and gently inflating it with fixative (preferentially formalin fixed tissue < 10% buffered at neutral pH) to avoid atelectatic changes [8]. Thus, before approaching a surgical lung biopsy, a multidisciplinary discussion with the entire panel of specialists, including expert bronchoscopists and/or thoracic surgeons, is crucial to make a better-informed diagnosis through the most adequate diagnostic approach.

Ancillary investigations (either on tissue or cytological samples) are crucial to support the diagnosis [4]. These will be mentioned in the sections below.

## New sources: liquid biopsy

In the landscape of occupational respiratory health, the integration of new biospecimens is progressively playing an important role in refining the diagnosis and in implementing predictive and prognostic biomarker exploration. “Biopsy” of biological fluids (namely liquid biopsy) (e.g., blood, urine, saliva/sputum, cerebrospinal fluid, pleural fluid, exhaled breath condensate, bronchoalveolar lavage fluid, ascites, or stool) is increasingly recognized as an efficient tool for non-invasive diagnosis, screening, and prognostication of several diseases, especially solid tumours [9–11]. In the setting of occupational lung diseases, the sputum and pleural fluid are biological matrices of particular interest that could become complementary to the other diagnostic tools.

*Sputum* is an easily obtainable specimen and may play an instrumental role in assessing occupational exposures by allowing the quantification of particulate matter, chemicals, or biological agents in various work environments. Anyway, the method of analysis needs to be better standardized [12].

In construction, it helps quantify respirable dust and particulate matter, revealing respiratory health risks. In chemical manufacturing, sputum analysis measures hazardous substance levels, guiding control measures [12]. Healthcare professionals benefit by quantifying exposure to airborne particles and chemicals [13]. Agricultural workers’ sputum analysis aids in understanding health risks from pesticides and allergens [14]. In metalworking, it evaluates the respiratory health impact of metal dust or fumes [15]. Methods such as mass spectrometry, chromatography (e.g., gas chromatography and high-performance liquid chromatography), and spectroscopy (e.g., infrared spectroscopy) are commonly employed [16]. These analytical approaches allow for the precise identification and quantification of specific substances, providing valuable insights into potential health hazards [16]. This quantitative aspect is fundamental not only for diagnosing diseases but also for understanding the extent of exposure, which is crucial for delineating occupational health risks. The application of such methods in this context poses technical challenges, primarily due to the scarcity of accredited laboratories possessing the requisite expertise and resources for precise analysis. This scarcity accentuates the critical need for meticulous selection and thorough scrutiny when submitting samples for examination. Collaborating with reputable laboratories that uphold stringent quality standards is paramount to guaranteeing the accuracy and validity of the generated data. Such partnerships are essential for advancing our understanding of occupational and industrial diseases, facilitating robust research outcomes, and ultimately enhancing public health interventions.

Moreover, some studies have explored the association between occupational exposures, such as exposure to airborne pollutants, dust, asbestos, or specific chemicals, and the presence of biomarkers in sputum samples, mainly to refine our ability to anticipate and address respiratory health challenges in diverse occupational settings [17, 18]. In these studies, researchers have focused on an array of biomarkers to discern early signs of lung disease or heightened risk. One avenue of investigation involves inflammatory markers, such as interleukins (e.g., IL-6 and IL-8), adhesion molecules (CD11b, CD35, CD163, and CD66), tumour necrosis factor-alpha (TNF-alpha), and C-reactive protein (CRP) [17, 18]. These markers serve as indicators of the body’s response to potentially harmful exposures. Genetic markers have also been of high interest, with a focus on polymorphisms in genes associated with respiratory function. Examples include genetic variations in genes like GSTM1, GSTT1, and SOD2, which may influence susceptibility to respiratory pathology or impact detoxification pathways [17]. Additionally, recent studies have explored the metabolome and microbiome alterations as potential biomarkers to inform on the severity of cellular damage and aid in environmental risk prevention [19].

In the context of occupational neoplastic lung diseases, especially mesothelioma, *pleural effusion* represents the worthiest of liquid biopsies. Soluble mesothelin-related peptides (SMRP), secreted glycoprotein, microRNAs, and CYFRA-21.1 are the most promising biomarkers being currently evaluated for diagnosis, prediction, and monitoring [20, 21]. These biomarkers have lower diagnostic capability but provide prognostic information with a potential role as therapeutic targets. A soluble mesothelin-related peptide (SMRP) is the only FDA-approved biomarker in patients with suspected mesothelioma [21]. With different serum and pleural fluid cutoffs, it can provide useful information in the diagnosis, prognosis, follow-up, and response to therapy in epithelioid mesothelioma.

It is beyond the scope of this manuscript to go into detail about these new tests, but it is extremely important to know that there is a growing interest by the specialists in this field to search for new liquid derived biomarkers because they require only minimally invasive sampling procedures, are valid contributors to a more comprehensive understanding of the disease, and help enhance diagnostic accuracy and monitoring capabilities.

In the next sections, we primarily describe the morphological characteristics of different occupational lung diseases as detectable by using the routine tools available in pathology laboratories, emphasizing their key pathological features and supportive analyses at the end of each section.

## Mineral dust diseases

### Silicates and other rare forms of dust-related diseases

*Silicatosis* is a lung condition caused by inhalation of silicates, which comprises silicates such as talc, mica, and kaolinite, as well as silicates such as fuller’s earth [4]*.* In general, silicates are less fibrogenic than silica. However, in prolonged and heavy exposure, and/or combined with silica, they can cause a fibrogenic pneumoconiosis. Clinical and functional features are similar to silicosis, whereas the radiographic opacities are more irregular. On morphology, there are no silicotic nodules, as the proportion of crystalline quartz in silica in the inspired dust is low (usually < 10%). The typical features comprise patchy and stellate-shaped centrilobular interstitial fibrosis (so-called “medusa head”) composed of a mixture of fibroblasts, collagen fibres, and dust-filled macrophages. There is abundant black carbon or brown iron dust mixed with numerous crystals with varying degrees of birefringence [22].

A special form of silicatosis is talc pneumoconiosis or *talcosis*. Indeed, talc is a frequent filler in medications used for oral consumption or may reach the lungs by the vascular route in drug abusers (intravenous injection of crushed tablets; in this case the talc granulomas are localized close to the pulmonary vasculature) [23]. At histology, focal peribronchial and perivascular fibrosis is associated with abundant dust deposits containing needle-shaped bluish-gray birefringent particles associated with giant cell response [23]. The number of macrophages and giant cells may be variable, and, in some cases, the granulomatous reaction is a close mimicker of sarcoidosis [23].

Other rare forms of dust-related diseases include *coal worker pneumoconiosis*, recently reported as an alarming resurgent pneumoconiosis [24]. Particulate matters directly interact with lung cells, leading to structural damage and the release of enzymes that contribute to lung scarring. Oxidative stress ensues as immune cells like alveolar macrophages scavenge particles, producing ROS and RNS that damage lipids, proteins, and DNA. This oxidative stress is exacerbated by the presence of heavy metals, transition metals, and polyaromatic hydrocarbons contaminants associated with the exposure. Moreover, inflammation and the production of growth factors are activated as lung cells release cytokines and growth factors in response to the exposure. These mediators recruit immune cells to the lungs, amplify inflammation, and activate fibroblasts, leading to the deposition of collagen and scarring of lung tissue. Genetic factors may also play a role in susceptibility to conditions like coal workers’ pneumoconiosis, with certain polymorphisms associated with increased risk and severity of the disease [25].

From the pathological point of view, two distinct forms are recognized: simple and complicated forms. The simple form comprises centrilobular macules, when associated with fibrosis, randomly distributed nodules in the upper lobes. The complicated form features are characterized by large and usually bilateral areas of fibrosis similar to progressive massive fibrosis. Progressive massive fibrosis is defined by the presence of a conglomerate of disease, at least 1cm in size, often associated with ischaemic necrosis, and associated with abundant dust laden macrophages with fibrosis. In both of them, there is an elevated black pigmentation that looks like the so-called “Medusa head”. The content of crystalline quartz, cristabolite, and tridymilite determines the pathological picture. Amongst anthracite coal workers, the high crystalline quartz provides a pathology similar to silicosis. For bituminous and lignite coal, with lower content quartz, the pathology is more that of a silicatosis. The pathologist can therefore provide information of likely prior exposure patterns. Nonetheless, birefringent particles may sometimes be detected within black areas, reflecting the mixed nature of coal dust. Emphysematous changes are typically present at the periphery of macules. Cavitation may occur as a complication of infection such as tuberculosis or ischemia due to vascular insult.

The most frequent occupational exposure of the above-described diseases is reported in Table 1.

**Table 1:** Occupational exposure profiles of silicates

Occupation	Exposure
*Construction worker*	Crystalline silica (quartz, cristobalite)
*Miner*	Silica dust (quartz)
*Glass manufacturing*	Amorphous silica, cristobalite, tridymite
*Ceramics industry*	Kaolin, talc, feldspar
*Demolition worker*	Crystalline silica (quartz)
*Agricultural worker*	Clay minerals, diatomaceous earth
*Foundry worker*	Silica dust, alumino-silicates
*Shipyard worker*	Crystalline silica, talc
*Quarry worker*	Crystalline silica (quartz)

#### Key morphological features

Silicatosis or other rare forms of dust: stellate-shaped centrilobular interstitial fibrosis composed of a mixture of fibroblasts, collagen fibres, and dust-filled macrophages. When nodules predominate, a diagnosis of silicosis should be made.

#### Supportive analyses

- *Polarized light microscopy*: birefringent crystals (silicates) in the centrilobular fibrotic brown areas of silicatosis.
- *Other investigations (used in referral centres)*: analytic electron microscopy and ion or laser microbe mass spectrometry (more frequently used for legal compensation).

### Silicosis

Silicosis is a fibrotic lung disease induced by inhalation of small particles (< 10 μm in diameter) of crystalline silica. Silica (silicon dioxide, SiO_2_) is a natural mineral, contained in more than 95% of the earth’s rocks. Historical and current occupational exposure are reported in Table 2 [24]. There are three different forms of silicosis whose clinical presentation is closely related to the cumulative dose of silica exposure [26]. Upon inhalation, freshly fractured silica particles trigger a complex cascade of pathological processes in the lungs. These particles can generate reactive free radicals on their surface, instigating DNA damage, mutations, and eventual cell death. Moreover, silica elicits a robust respiratory burst in lung macrophages, akin to the response seen with asbestos fibres, resulting in the release of oxidants, proteolytic enzymes, and pro-inflammatory cytokines such as TNF-α and IL-1β. This inflammatory milieu orchestrates the recruitment of additional inflammatory cells, perpetuating severe pulmonary inflammation.

**Table 2:** Historical and current occupational exposure to silica

Historical occupational exposure	Current occupational exposure
*Mining, quarrying, and stone cutting*	Denim sandblasting
*Foundry work, glass manufacturing, and tunneling*	Hydraulic fracturing to extract natural gas or oil
*Construction, shipbuilding, and abrasive blasting*	Cutting of engineered/artificial stones

**Fig. 1:**
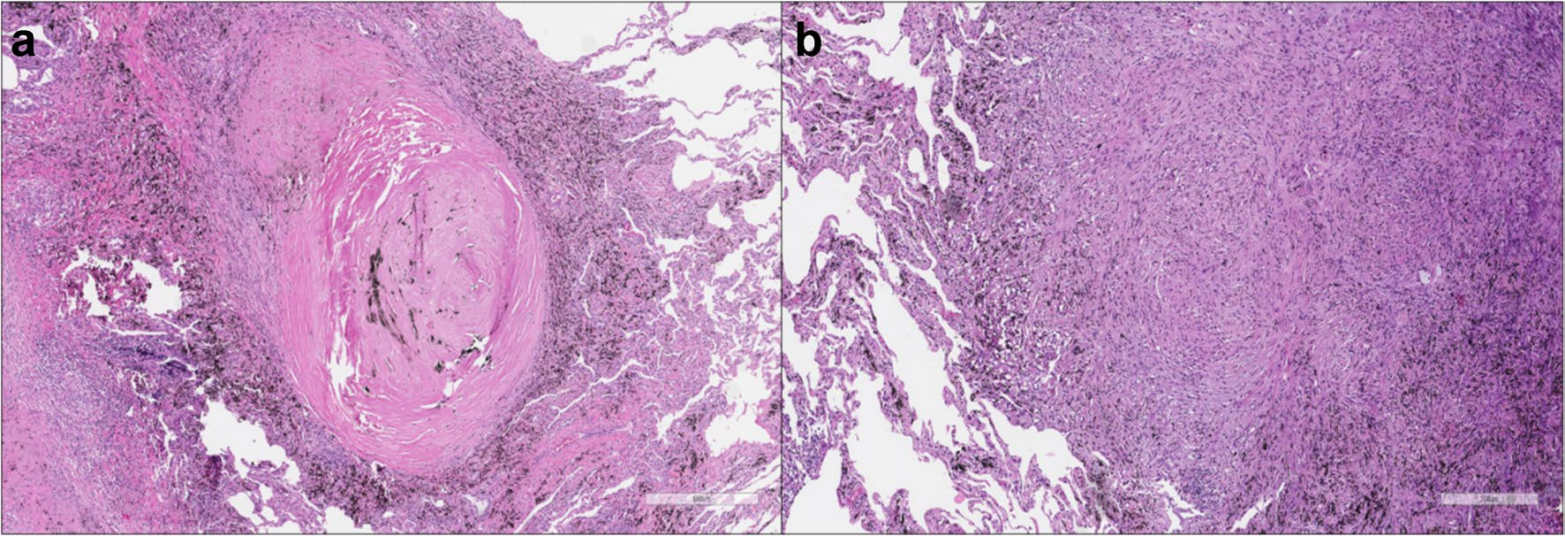
Silicotic Nodules. **a** The nodules exhibit a distinct morphology characterized by sharply circumscribed structures with whorled, densely hyalinized collagen (haematoxylin and eosin, scale bar: 500 µm). **b** In the early stages of lesion formation, macrophages envelop the fibrotic centre, contributing to the nodular structure (haematoxylin and eosin, scale bar: 300 µm)

Of note, chronic silicosis has a latency of 10 or more years of low to moderate exposure dose; accelerated silicosis occurs within 10 years of moderate to high levels of exposure, and acute silicosis, which is associated with a high concentration of crystalline silica, may occur within a week to 5 years from the initial contact [27]. Chronic silicosis presents as simple or nodular silicosis, which features fibrotic nodules (< 1 cm in diameter), usually in the upper lobes. The typical silicotic nodules appear as sharply circumscribed nodules consisting of whorled, densely hyalinized collagen. In the recently formed lesions, macrophages form a mantle around the fibrotic centre (Fig. 1).

Calcification and ossification may be detected in long-standing lesions. Examination with polarizing microscopy shows birefringent particulate within the fibrotic nodules. Larger brightly birefringent particles, which represent silicates or silicates-like particles, may also be detected. Silicotic nodules may also protrude through the visceral pleura. The nodules may merge to form fibrotic conglomerates (> 1 cm) typical of complicated or progressive massive fibrosis (PMF) [28, 29]. The pathological definition of PMF is still debated: for PMF to be correctly diagnosed, some have proposed an extension of at least 2 cm in any single dimension of tissue while others report that it should be at least 1 cm**.** Additional findings are enlarged mediastinal lymph nodes with nodular fibrosis and peripheral calcification (present in 75% of silicosis patients) [30, 31]. Acute and accelerated PMF has been described to occur in high-concentration exposure workers with a short latency. Acute silicosis or silico-proteinosis shows features of alveolar proteinosis, and accelerated silicosis may present fibrotic nodules and masses characteristic of PMF [26]. The granular eosinophilic material filling the alveoli is strongly positive to staining with periodic acid-Schiff-diastase (PAS-D). Conditions associated with silicosis comprise immunological disorders such as polyclonal hyper-gammaglobulinemia, increased rheumatoid factor or antinuclear antibodies, systemic sclerosis, and rheumatoid disease. Among all infective complications, tuberculosis is the most frequent infective complication [4]. Crystalline silica, particularly quartz, is also recognized as a potent carcinogen, classified as Group 1 by the International Agency for Research on Cancer (IARC). This classification is based on extensive evidence from epidemiological studies, animal research, and mechanistic studies demonstrating its association with lung cancer. Inhalation of quartz particles leads to the generation of ROS and RNS in the lung tissue, causing oxidative stress, DNA damage, and inflammation [25]. On the other hand, amorphous silica, lacking the crystalline structure of quartz, is generally considered to have a lower carcinogenic potential. While both forms of silica can cause respiratory diseases such as silicosis and fibrosis, epidemiological and experimental evidence suggests that amorphous silica is less likely to induce lung cancer. Regulatory agencies and health organizations typically classify amorphous silica as a lower hazard compared to crystalline silica in terms of carcinogenicity, reflected in occupational exposure limits and safety guidelines [32].

The most frequent histological differential diagnoses are reported in Table 3.

**Table 3:** Most frequent histological differential diagnoses of silicosis

Differential diagnosis	Histological features
*Chronic beryllium disease*	Chronic beryllium disease (CBD) | granulomas with central necrosis, lymphocytic infiltrates
*Asbestosis*	Interstitial fibrosis, asbestos bodies
*Coal worker’s pneumoconiosis*	Coal macules, progressive massive fibrosis
*Sarcoidosis*	Non-caseating granulomas, lymphocytic infiltrate
*Tuberculosis*	Caseating granulomas, central necrosis, inflammatory cells
*Welders’ lung pneumoconiosis*	Siderosis is mainly characterized by dusts macules containing coarse and brown-black deposits
*Rheumatoid nodules*	Nodules with necrotic centre, surrounded by palisading histiocytes, plasma cells, and lymphocytes
*Malignancy*	Occasionally, lung nodules identified in radiology may represent metastatic deposits if multiple nodules are noted
*Pulmonary Langerhans cell histiocytosis*	Centrilobular inflammatory nodules with eosinophils, dendritic, and Langerhans cells, intra-alveolar smoke exposure pigment-laden macrophages in early stages; may evolve to interstitial fibrosis

#### Key morphological features

- Chronic silicosis:
  - Nodules: discrete, black-pigmented fibrotic nodules, < 1 cm in diameter, frequently localized around respiratory bronchioles, small pulmonary arteries, and lymph nodes.
  - These nodules may coalesce in advanced stages giving the appearance of progressive massive fibrosis.
- Acute silicosis:
  - Features typical of alveolar proteinosis (silico-proteinosis): granular eosinophilic material (strongly positive PAS/PAS-D).

#### Supportive analyses

- *Polarized light microscopy*: faintly birefringent particles within fibrotic nodules, which are not as extensive as in silicatosis.
- *Special stains or molecular investigation for microorganisms* especially mycobacteria in case of cavitated nodules (so-called: silico-tuberculosis).
- *Other investigations (used in referral centres)*: analytic electron microscopy and ion or laser microbe mass spectrometry (more frequently used for legal compensation).

### Metal-induced lung diseases

Inhalation of metallic dust can cause different pulmonary diseases subject to host factors. While heavy metals encompass a broad spectrum of elements with diverse properties and potential health impacts, the focus in many reviews often centres on a select few metals. This selective attention is often driven by several factors, including the prevalence of certain metals in industrial processes, their widespread environmental distribution, and their documented association with significant health effects. Here we consider the most common pathological features: siderosis, aluminosis, berylliosis, and hard-metal disease.

Historical and current occupational exposure is reported in Table 4. *Siderosis* does not display a lung tissue reaction on exposure to iron oxide particles with only intrapulmonary pigment deposition appreciated. Iron is an inert metal with no pathogenicity to lung tissue. Usually, asymptomatic patients have no functional impairment, but imaging studies as well as histopathology show nodular accumulation characterized by macrophages loaded with yellow brown globules with dark centres [33]. A good example of siderosis is observed in arc welders. Fibrosis may be seen in forms with concomitant exposure to other fibrogenic dust such as in sidero-silicosis.

**Table 4:** Occupational exposure profiles of metals/hard metals

Occupation	Exposure
*Mining and metallurgy*	Lead, mercury, arsenic High exposure due to processes
*Metalworking*	Chromium, nickel, manganese Fumes from welding and cutting
*Hard metal industries*	Materials: cobalt, tungsten Exposure during manufacturing

*Aluminosis* is a rare disease caused by the inhalation of aluminium-containing dust. Aluminium is a lightweight metal widely used in industry; hypersensitivity to aluminium is believed to play a role in the pathogenesis of the disease [34]. The tissue reaction is variable from grayish dust-laden macrophages accumulated in perivascular and peribronchiolar areas to granulomatous inflammation, and in rare instances leading to interstitial fibrosis. The aluminium dust is refractile. Rare cases of alveolar proteinosis, similar to that seen acute silico-proteinosis, have also been reported [33].

Berylliosis is caused by the inhalation of beryllium containing dust. It induces diffused alveolar damage in the acute form, and non-necrotizing sarcoid-like granulomas in the chronic form with bilateral lymphadenopathy. Granulomas can sometimes show multinucleated giant cells with Schaumann and asteroid bodies. Pathogenetic mechanism is most likely a delayed hypersensitivity reaction associated with certain HLA haplotypes [35]. The differential diagnosis from sarcoidosis is impossible without clinical information or an environmental exposure history. Ancillary methods like positive beryllium lymphocyte proliferation test may be helpful in rendering correct diagnosis.

*Hard metal lung disease*: Hard-metal lung disease (HMLD) represents a hypersensitivity reaction caused by exposure to inhalation of hard-metal particles, whose major components are tungsten carbide (approximately 90%) and cobalt (approximately 10%) [36]. HMLD often manifests as giant cell interstitial pneumonia characterized by fibrotic thickening of alveolar septa accompanied by a mild/moderate chronic inflammatory infiltrate. Multinucleated giant cells, which are often bizarre cells, are a frequent feature and are found within both the alveolar space and lining of the alveolar septa. Giant cells can show features of emperipolesis. Heavy metals are naturally occurring elements characterized by their high atomic weight and density. The toxicity of heavy metals is influenced by several factors, including the amount of exposure, the route of exposure, chemical form, as well as individual factors such as age, gender, genetics, and nutritional status. Among the prioritized metals with significant public health implications are arsenic, cadmium, chromium, lead, and mercury. In this context, cobalt merits particular attention due to its prevalent use in hard metal alloys. While cobalt is not the only metal of concern, it is one of the major components found in hard metal alloys and has been associated with various health risks, including respiratory issues and dermatological reactions.

**Fig. 2:**
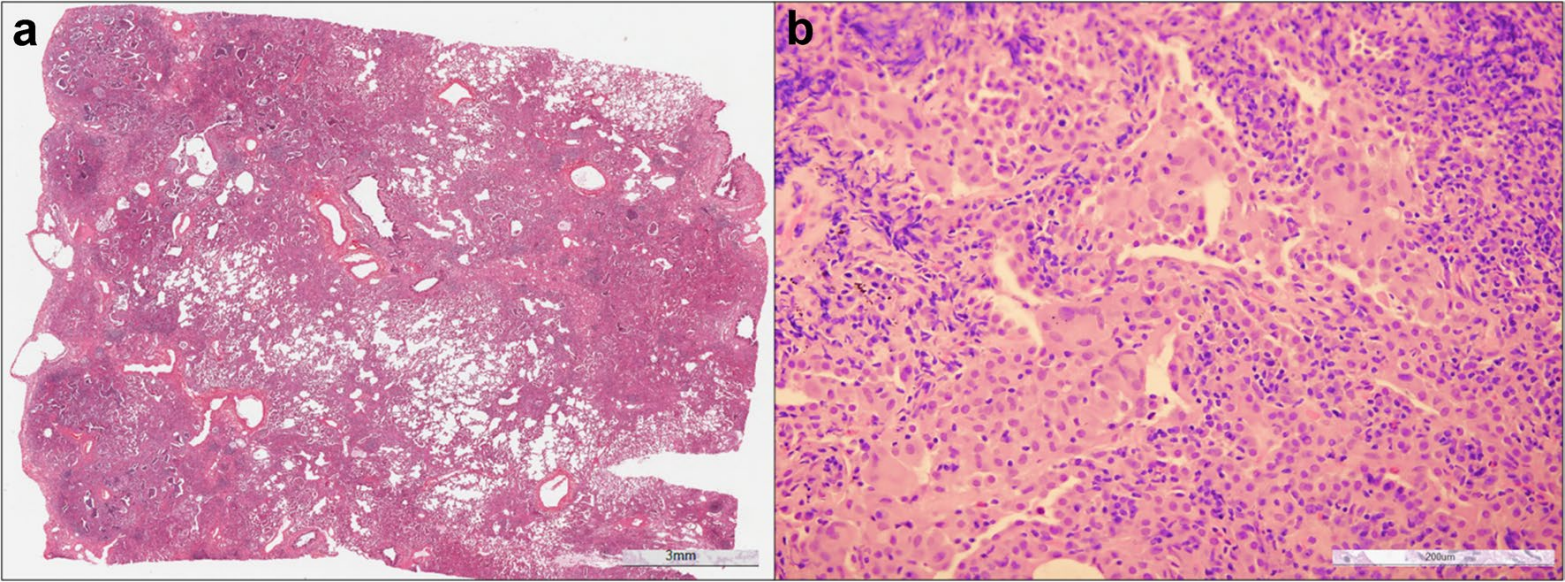
Pathological features in hard metal disease lung tissue. **a** Occasional features reminiscent of usual interstitial pneumonia (UIP)-like areas, characterized by microscopic “honeycomb” patterns indicative of fibrosis, can be detected (haematoxylin and eosin, scale bar: 3 mm). **b** Additionally, some macrophagic alveolitis with DIP-like features and several multinucleated giant cells are observed (haematoxylin and eosin, scale bar: 200 µm)

Causative metal is cobalt which is difficult to detect because of water solubility. Initially, it elicits fibrosis in the airway wall, which clinically manifests as small airway disease. It is estimated that only 1% of exposed individuals develop interstitial lung diseases, while asthma is diagnosed in 10% of exposed cases (Fig. 2).

The most frequent histological differential diagnoses of the above-reported metal and hard-metal diseases are reported in Table 5.

**Table 5:** Most frequent histological differential diagnoses for metal/hard metal diseases

Differential diagnosis	Histological features
*Hypersensitivity pneumonitis*	Inflammatory infiltrates, non-caseating granulomas
*Granulomatous lung diseases*	Formation of granulomas with varying compositions
*Occupational lung diseases*	Variable, depending on specific occupational exposure

#### Key morphological features

- Siderosis: peri-bronchial/perivascular deposition of dark brown to black material (iron oxide).
- Aluminosis: peri-bronchial/perivascular deposition of refractile gray dust.
- Berylliosis: well-formed non-necrotizing granulomas.
- HMLD: giant cell interstitial pneumonia and multinucleated giant cells with emperipolesis.

#### Supportive analyses

- *Special stains:* iron stain useful in siderosis, and Irwin’s aluminon in aluminosis.
- *Polarizing microscopy*: negative for all these dust particles with no birefringence detected.
- *Other investigations (used in referral centres)*: analytic electron microscopy, ion or laser microbe mass spectrometry, and wet chemical analyses, particularly useful in berylliosis, beryllium lymphocyte proliferation test.

### Asbestos-related lung diseases

Asbestos is a collective term that defines a regulated group of six naturally occurring, highly fibrous silicate minerals. The six minerals fall into two groups: serpentine, of which the only asbestiform type is chrysotile (white asbestos), and amphibole asbestos, which comprises the commercial forms of asbestos—amosite (brown) and crocidolite (blue)—as well as the non-commercial forms: asbestiform tremolite, asbestiform anthophyllite, and asbestiform actinolite. The two groups—amphibole asbestos and serpentine chrysotile—have different physical, chemical, and biological properties. The propensity of asbestos minerals to induce disease correlates closely with the retention of biopersistent fibres in the body. Amphibole forms of asbestos are far more potent in the induction of disease than chrysotile. Although in the western world, the levels of exposure are currently kept in check by strict regulations, past asbestos exposure continues to affect many due to the latent nature of the pathophysiological response of the body to the inhaled fibres. Historical and current occupational exposures are reported in Table 6.

**Table 6:** Occupational exposure profiles of asbestos

Occupation	Exposure
*Construction and building trades*	High levels due to the widespread use of asbestos| in building materials, insulation, and roofing
*Shipbuilding*	Asbestos used extensively in ship construction, leading to high exposure levels
*Textile mills*	Asbestos used in textiles, especially for fire-resistant clothing, leading to inhalation risks
*Automotive industry*	Asbestos used in brake linings and clutch components, posing a risk during maintenance and repairs

In asbestosis, there is a significant influx of macrophages into the lung tissue, which triggers an upregulation of inflammatory mediators such as TNF and interleukins (IL-1β and IL-6) [37]. This inflammatory response is further intensified by the heightened generation of ROS. These inflammatory processes, coupled with increased ROS levels, contribute to the destruction of alveolar type 1 cells, impairing their ability to facilitate gas exchange. Concurrently, the deposition of asbestos fibres results in the formation of characteristic asbestos and ferruginous bodies. These bodies, composed of asbestos fibres enveloped in iron-containing proteins, serve as distinctive markers of chronic asbestos exposure. The toxicity of mineral fibres is influenced by various factors, including chemical composition, surface reactivity, crystallinity, and the presence of transition metals. Additionally, fibre size and shape impact their ability to penetrate the alveolar space and provoke an inflammatory response. Mechanistically, frustrated phagocytosis of longer asbestos fibres by alveolar macrophages leads to chronic inflammation, activating the NLRP3 inflammasome and promoting malignant transformation. The WHO defines asbestos based not only on mineralogy but also on fibre dimensions. The NLRP3 inflammasome responds to diverse stimuli, including asbestos and carbon nanotubes, inducing granulomatous inflammation. Scavenger receptors, particularly MARCO and SR-B1, play a role in recognizing and mediating the cellular uptake of asbestos fibres, contributing to pulmonary fibrosis and asbestos-related diseases.

Indeed, long asbestos fibres, which persist in the pleura, trigger prolonged inflammation due to frustrated phagocytosis, leading to the activation of the NLRP3 inflammasome and the release of inflammatory cytokines like IL-1ß. This chronic inflammatory state generates ROS and RNS capable of causing DNA damage. Additionally, the presence of excess iron, associated with asbestos bodies further exacerbates carcinogenesis. Mutations in the BAP1 gene, observed in a significant proportion of mesotheliomas, play a crucial role in suppressing cell death mechanisms. BAP1 mutations enable mesothelial cells to evade apoptosis and accumulate further DNA damage, leading to carcinogenesis. The BAP1 protein regulates DNA repair and apoptosis following DNA damage, and cells with reduced BAP1 activity are less susceptible to cell death processes like ferroptosis. Furthermore, HMGB1 released during cell necrosis promotes autophagy and suppresses apoptosis, facilitating the accumulation of mutations associated with carcinogenesis. In summary, asbestos creates a mutagenic microenvironment rich in ROS and HMGB1, promoting DNA damage in mesothelial cells. Subsequent mutations, particularly in genes like CDKN2A, NF2, and TP53, accumulate due to the impaired cell death mechanisms mediated by BAP1 mutations. As consequence, the adverse effects of asbestos generally fall under two major categories: non-neoplastic (pleural thickening, effusion and plaque, and asbestosis) and neoplastic thoracic disease (mesothelioma and lung carcinoma).

Here, we particularly emphasize the pathological features of asbestosis, mesothelioma, and lung carcinoma.

#### Asbestosis

*Asbestosis* is defined as diffuse pulmonary fibrosis due to a prolonged cumulative inhalation of massive doses of asbestos fibres with a positive correlation between the number of asbestos fibres and the severity of the disease. The latency period is approximately 15 years but could be longer. The disease may continue to progress even if occupational exposure has ceased.

In 2010, a joint report by the Asbestosis Committee of the College of American Pathologists and the Pulmonary Pathology Society published by Victor Roggli et al. [38], updated the diagnostic criteria, which was followed by an update in 2016 [39], responding to critical issues addressed after the initial publication. The diagnosis is usually based on (1) previous history of exposure to any type of asbestos fibres (AF), (2) clinical findings, (3) CT scan showing reticular linear diffuse opacities in the lower lobes, (4) restrictive alterations of lung functions and alteration in CO transfer, and (5) in some cases hyaline fibrous plaque or diffuse pleural fibrosis. Criteria 1 and 3 are mandatory for clinical diagnosis in symptomatic patients. However, even in the second half of the twentieth century, many cases of asbestosis remain asymptomatic. In asymptomatic patients, histologic assessment is required when the context of AF is equivocal when the clinical or radiologic features are atypical in the case of associated lung cancer, or at autopsy for medico-legal purposes. The disease is more severe in the sub-pleural regions and lower lobes.

A pathologist is required to identify both the pattern of interstitial lung fibrosis—in asbestosis it is defined as acellular and collagenous rather than fibroblastic and inflammatory, plus the presence of necessary biomarkers of asbestos exposure—either asbestos bodies determined by light microscopy, or asbestos fibres defined by mineral analysis. Attanoos [40] showed that the pattern of lung fibrosis in asbestosis is one best regarded as a fibrotic non-specific interstitial pneumonia (NSIP) with subpleural accentuation and fibroelastotic degeneration rather than a usual interstitial pneumonia. The presence of UIP should alert the pathologist to a causation other than asbestos, such as idiopathic pulmonary fibrosis, chronic hypersensitivity pneumonitis, or collagen vascular disease. The asbestos body count requires the presence of two asbestos bodies (AB) (rod-like beaded or dumbbell-shaped structures with a thin translucent core) per square centimetre on fresh or formalin-fixed tissue biopsy samples (from the peripheral lower lobe) to exclude the diagnosis of fibrotic NSIP with subpleural accentuation. Cryobiopsy has recently been found to allow for larger specimens compared to transbronchial biopsy which should not be performed in this setting except in exceptional circumstances. BAL containing > 1 AB/ml indicates a high probability of AE [41].

**Fig. 3:**
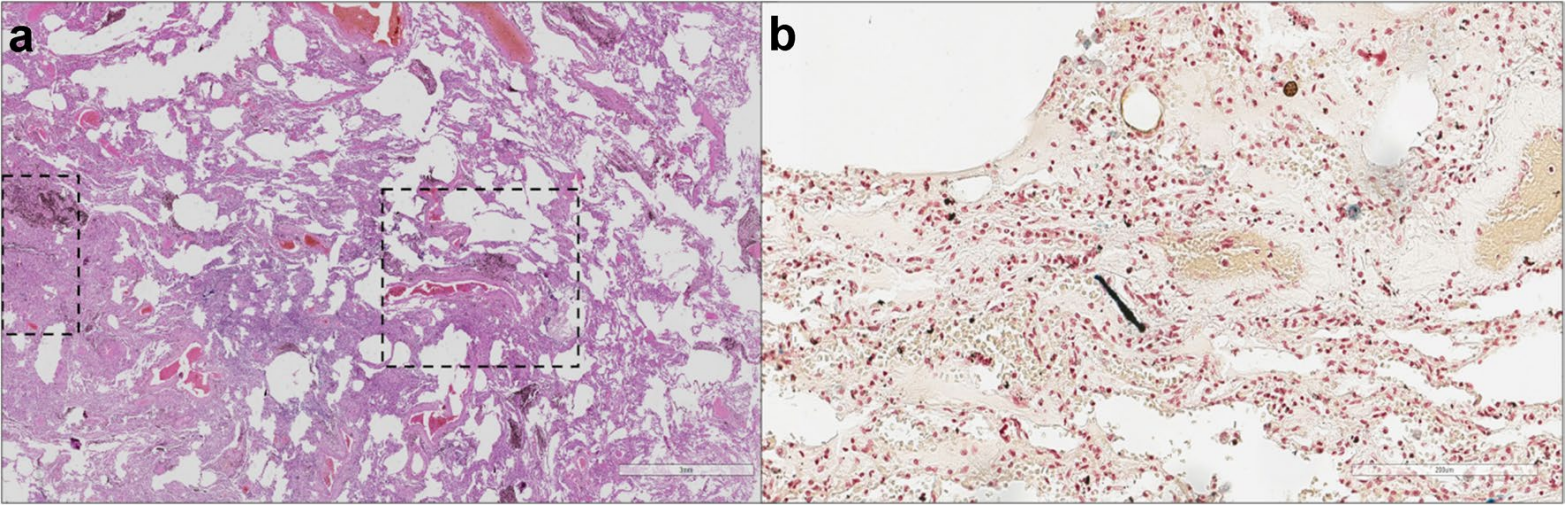
Histopathological characteristics of asbestosis. **a** Lung tissue from a patient with asbestosis with a fibrotic thickening of two close respiratory bronchi (dotted lines), extended to some alveoli (haematoxylin and eosin staining, scale bar: 3 mm). **b** Microscopic examination using Perls stain highlighted an asbestos body (Perls Prussian blue stain, scale bar: 200 µm)

Histological features include diffuse fibrosis, which is characteristically paucicellular, more collagenous than fibroblastic, with lack of inflammation, numerous intra alveolar macrophages, most severe at the periphery of the lung with some degree of pleural fibrosis, and commonly with pleural plaques. Fibroblastic foci are very uncommon (Fig. 3).

In early asbestosis, the grading scheme proposed by Roggli et al. [38] is subdivided into four grades (see table 7). Initially, the fibrosis is limited to the wall of the alveoli around the bronchioles. However, small airway disease (≤ 2 mm) is still a matter of debate [42]. To avoid confusion with smoking bronchiolitis, in 2016 [39], the committee decided that patients with only bronchiolar wall fibrosis have obstructive disease rather than asbestosis. Accelerated asbestosis may occur with a fibrotic lung without honeycombing in association with numerous AB caused only by amphiboles.

**Table 7:** Histologic grading scheme according to Roggli and Sporn modified 2010–2016 from the scheme presented by Craighead et al. (Archi Pathol Lab Med 1982)

Grade	Description
*Grade 0*	No appreciable peribronchiolar fibrosis or fibrosis confined to the bronchiolar walls
Grade 1*	Fibrosis confined to the walls of respiratory bronchioles and to the first tier of adjacent alveoli
Grade 2*	Extension of fibrosis to involve alveolar ducts and/or ≥ 2 tier of alveoli adjacent to the respiratory bronchiole
*Grade 3*	Fibrosis thickening of the walls of all alveoli between ≥ 2 adjacent respiratory bronchioles
*Grade 4*	Honeycomb changes

^*^Recommendations from the authors: *Grade 1 and 2 should be distinguished from smoking-induced peribronchial fibrosis or other mixed dust pneumoconiosis*

##### Key morphological features

Key features with the respective grading score are reported in Table 7. However, the hallmark of the disease is the occurrence of asbestos bodies (at least 2/cm^2^).

##### Supportive analyses

- *Special stains:* iron stain is useful since it may facilitate the detection of asbestos bodies (deep blue colour).
- *Polarizing microscopy*: not useful.
- *Other investigations (used in referral centres)*: analytic electron microscopy. Mineral analysis may be conducted by various methods—light microscopic methods are now recognized as of limited utility as they cannot characterize minerals and do not detect most small particulates. Electron microscopy is useful in determining disease diagnosis and disease causation—it has particular use when the exposure history is not clear.

#### Mesothelioma

**Fig. 4:**
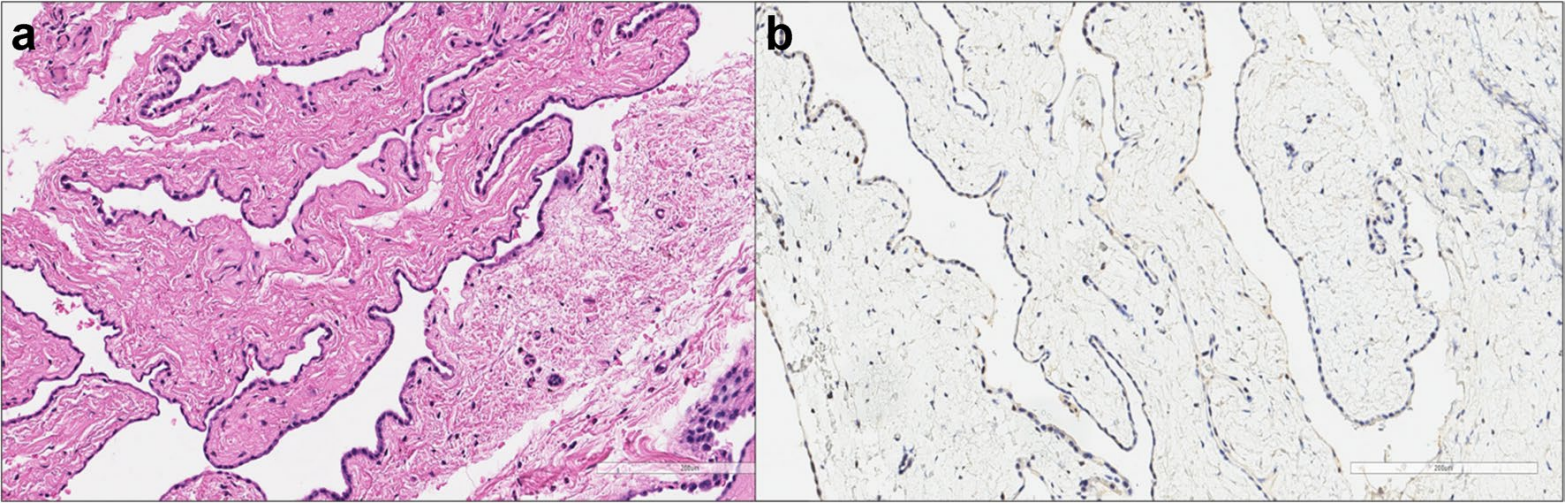
In situ mesothelioma. **a** The image shows the abnormal growth of neoplastic mesothelial cells in a single layer (haematoxylin and eosin, scale bar: 200 µm). **b** The immunohistochemical staining pattern demonstrates a notable loss of BAP1 expression (BAP-1 immunohistochemistry, scale bar: 200 µm)

Among asbestos-related diseases, mesothelioma is certainly the most common. Diffuse mesothelioma is the most frequent primary neoplasm of the pleura, strongly associated with occupational exposure to commercial amphibole asbestos in more than 80% of men and 20–40% of women after latency period of 30–40 years. Other rare causes include different mineral fibres, such as erionite, and therapeutic and occupational radiation, with mesothelioma development occurring only 10 years after exposure [43]. Amongst patients with mesothelioma with no clear external exposures, there is a clear recognition that these arise through internal genetic mechanisms associated with age and cumulative DNA replicative mutations. Germline mutations with pathogenic variants are found in 12% of patients, with 25% of those exhibiting the BRCA1-associated protein-1 (BAP1) predisposition syndrome [44]. This group is reported to have an increased risk of developing mesothelioma at a younger age. Subjects with mesothelioma associated with inherited genetic syndromes are considered more likely in those < 50 years age; in those with peritoneal site disease; epithelioid histology, low grade with high inflammatory tumour microenvironment pathology, a personal history of cancer, and a family history of cancer. Amongst young subjects, especially with peritoneal mesothelioma, there is a requirement for testing for specific genetic fusions. The fifth edition of the 2021 WHO classification [45] has changed the terminology of malignant mesothelioma to mesothelioma (“malignant” is no longer recommended) and has maintained the subdivision into three major subtypes (epithelioid, biphasic, and sarcomatoid) regardless of whether the tumour is diffuse or localized. The most significant changes of the fifth WHO classification is the identification of mesothelioma in situ (MIS) in the clinical context of an unresolving pleural effusion with no radiological or thoracoscopic evidence of tumour. MIS is described as a pre-invasive single-layer surface proliferation of neoplastic mesothelial cells showing either BAP1 nuclear loss or MTAP cytoplasmic loss by immunostaining a surrogate of *CDKN2A* homozygous deletion by fluorescence in situ hybridization assay (Fig. 4).

The diagnosis cannot be made on cytology alone and needs a minimum of 100 to 200 mm^2^ of biopsy tissue sample. A multidisciplinary approach is essential [45]. Recently, Galateau Salle et al. reported a series of MISs mimicking well-differentiated papillary mesothelial tumours (WDPMT) showing BAP1 loss, supporting the decision to change the terminology of well-differentiated papillary mesothelioma in WDPMT to avoid confusion and misdiagnosis for treatment and medico-legal purposes [46]. Indeed, WDPMT is a single or multifocal papillary lesion, with retained BAP1 and absence of *CDKN2A* homozygous deletion lesion characterized by a slowly growing disease, usually without relation to asbestos exposure [45]. Although mesothelioma is a particularly aggressive tumour, with a survival of nearly 12 months without treatment, and with less than 5% of patients alive at 5 years, the prognostic value of histopathological subtypes impacts treatment decisions and surgery options. Epithelioid mesothelioma (EM) is the most frequent subtype (~ 80%) with a slightly better survival compared to the highly aggressive sarcomatoid mesothelioma (SM) (7%) which is drug resistant, and the biphasic subtypes (13%) with survival usually being dependent on the percentage of the sarcomatoid component [47].

At histology, a major effort has been made to better stratify the histopathologic characteristics divided into architectural pattern, cytological, and stromal features according to prognostication (Table 8) [47]. The key histological features of epithelioid subtypes of poor prognosis are solid and micropapillary, architectural pattern, and highly aggressive pleomorphic cytological features (large anaplastic and giant cells and bizarre nuclei), while EM with myxoid stroma and < 50% of solid component support the best prognosis. Lympho-histiocytoid mesotheliomas that may mimic lymphoma or lymphoepithelial carcinomas are also indicators of a better prognosis. Grading has emerged as an important prognostic factor only for epithelioid subtype and a two-tiered grading system (low versus high grade) based on atypia; mitotic count and necrosis have been integrated in the fifth WHO edition (Table 9) [45, 47]. SM is highly heterogeneous from bland to highly atypical, with high mitotic count and massive necrosis. Desmoplastic mesothelioma is characterized by abrupt demarcation between spindle cells and dense hyalinized stroma of more than 50% of the proliferation mimicking pleural plaque. Other features include the presence of heterologous elements (rhabdomyosarcomatous, osteosarcomatous, and chondrosarcomatous which is to be distinguished from osteoid metaplasia), with pleomorphic and transitional cytological features [45] being previously described as epithelioid pattern. The transitional pattern of mesothelioma has been defined in the WHO classification as a feature of epithelioid malignant mesothelioma showing a sheet-like growth pattern in which cells are cohesive, with elongated morphology, not overtly spindle-shaped, and lacking distinct sarcomatous features [48, 49].

**Table 8:** Organization of mesothelioma characteristics according to the WHO fifth classification [42]

Subtype	Architectural pattern/cytological features/stromal characteristics	Outcomes prognosis	Additional reporting
Epithelioid (~ 80%)	**Architectural pattern**		% of each architectural pattern on surgical or EPP specimen
	Tubulopapillary	Better prognosis	
	Trabecular	Better prognosis	
	Adenomatoid	Better prognosis	
	Solid	Poor prognosis	
	Micropapillary	Poor prognosis	
	**Cytological features**		
	Rhabdoid	Not known	
	Deciduoid	Not known	
	Small cell	Not known	
	Clear cell	Not known	
	Signet ring cell	Not known	
	Lymphohistiocytoid	Better	
	**Stromal features**		
	Abundant myxoid stroma with low solid component < 50%	Best prognosis	
	**Grade**		
	Low grade	Best prognosis	
	High grade	Worst	
Sarcomatoid (7%)	**Architectural pattern**		
	None		
	**Cytological features**		
	Lymphohistiocytoid	Better prognosis	
	Pleomorphic	Worst	
	Transitional	Worst	
	**Stromal features**		
	Desmoplastic	Usually, poor prognosis	
	With heterologous elements	Not known	
Biphasic (13%)	% f sarcomatoid component		
	- 80%	Poor prognosis	
	- With transitional features	Worst

**Table 9:** Nuclear grading for epithelioid mesothelioma according to the WHO fifth classification [42]

• Nuclear grade	• 1 = mild, 2 = moderate, 3 = severe
• Mitotic count score	• 1 = < 1 mitose/mm^2^, 2 = 2–4 mitosis/mm^2^, 3 ≥ 5 mitoses /mm^2^
• Total nuclear grade score	• Nuclear grade I = 2 or 3, nuclear grade II = 4 or 5, nuclear grade III = > 6
• Necrosis	• Absent of present
• Final tumour grade	
- Low grade	• Nuclear grade I or II without necrosis
- High grade	• Nuclear grade II with necrosis and grade III with or without necrosis

Galateau-Salle et al. showed that reticulin staining helps separate epithelioid from transitional morphology and that the genomic events of transitional mesothelioma are similar to the SM subtype, usually associated with a very dismal prognosis. Biphasic mesothelioma consists of a mix of the epithelioid and sarcomatoid components, the latter of which should be reported for potential implication in prognosis and therapeutic management. Should EM be combined with a transitional component, the tumour would be considered biphasic.

**Fig. 5:**
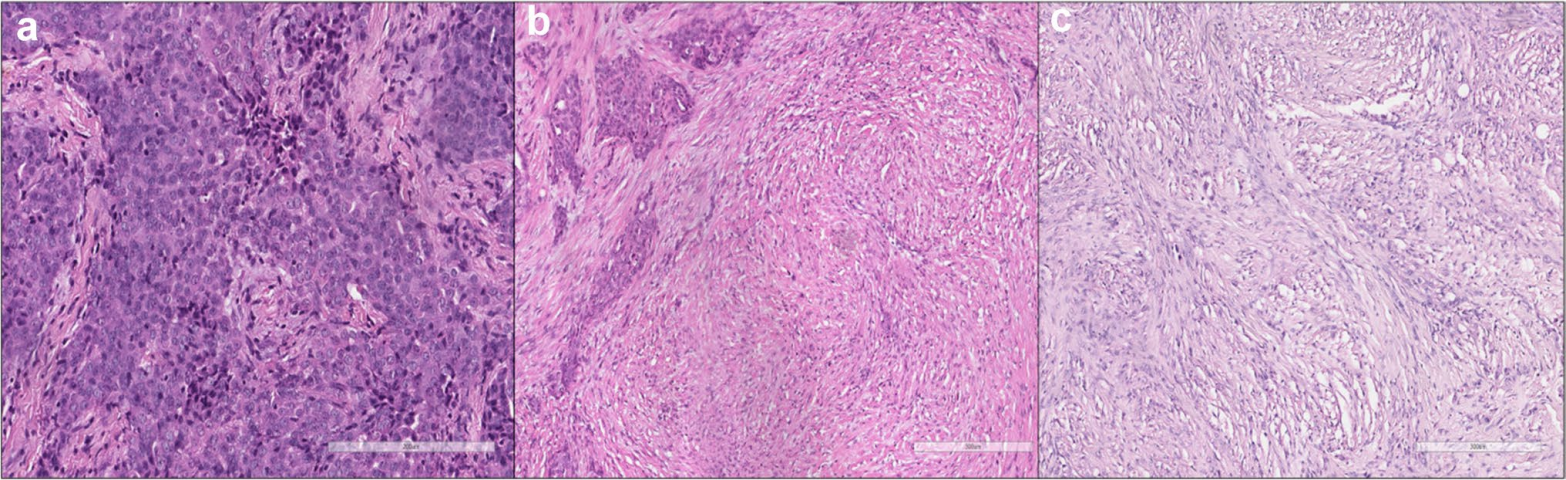
Mesothelioma histotypes. In this figure, the three primary histotypes of mesothelioma are highlighted. Specifically, it includes **a** the epithelioid solid type graded as Grade 2 (haematoxylin and eosin staining, scale bar: 200 µm), **b** the biphasic subtype (haematoxylin and eosin staining, scale bar: 300 µm), and **c** the sarcomatoid variant (haematoxylin and eosin staining, scale bar: 300 µm)

**Fig. 6:**
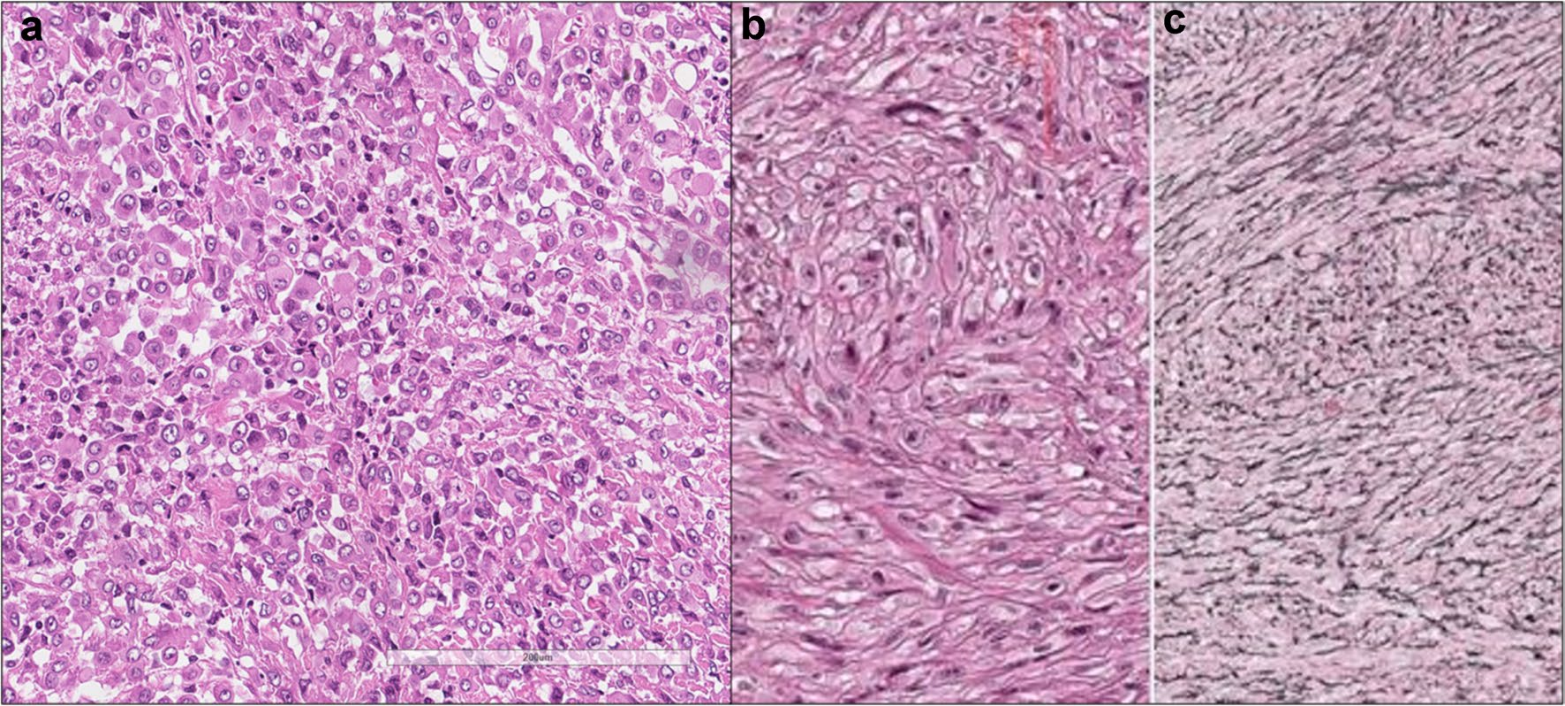
Histological prognostic details. **a** The presence of large bizarre anaplastic cells/giant cells, and **b** the transitional pattern, characterized by a sheet-like arrangement of elongated cells with well-defined borders, is indicative of a poor prognosis. Notably, the transitional pattern can be more distinctly visualized by reticulin staining (**c**) (**a, b, c**: haematoxylin and eosin staining, scale bar: 200 µm)

Figure 5 illustrates the distinctive features of solid epithelioid, biphasic, and sarcomatoid mesotheliomas (a, b, c), alongside certain histological characteristics associated with a poorer prognosis (Fig. 6).

##### Key morphological features

Key features concerning architectural pattern/cytological features/stromal characteristics are reported in Table 8**.**

##### Supportive analyses

- Immunohistochemistry: essential in all subtypes of mesothelioma for diagnosis. The antibodies used as mesothelial markers include: Calretinin, WT1, D2-40, and CK 5/6. GATA 3 is positive in approximately 70% of sarcomatoid mesotheliomas, very useful for the differential diagnosis of metastatic pulmonary sarcomatoid carcinoma [50]. Many algorithms have been published using positive mesothelial markers and negative (or organ-specific) markers [47, 48]. HEG1 and Claudin 4 are two immunohistochemical markers to separate EM from carcinomas with a high sensitivity and specificity, respectively, of 99.7% and 98.9% in favour of EM [48]. Loss of BAP1 and MTAP can support the diagnosis of mesothelioma versus benign mesothelial proliferation: however, morphology (with an invasive pattern) is still the essential tool to distinguish malignant from benign disease.
- Molecular analyses: FISH for alterations in *CDKN2A* and *NF2* (as IHC for BAP1 and MTAP) can be used to support the diagnosis of mesothelioma versus benign mesothelial proliferation. Comprehensive genomic and transcriptomic sequencing revealed major genomic intra and inter-tumour heterogeneity among patients [49]. However, molecular testing for predictive biomarkers of response to systemic therapies is not yet recommended. The most frequent genomic alterations are *BAP1*, *CDKN2A*, *NF2*, followed by *TP53*, *SETD2*, *DDX3X*, *ULK2*, *RYR2*, *CFAP45*, *SETDB1*, and *DDX51*. BAP1 IHC loss is present in ~ 60% of pleural mesothelioma (PM) and should be at least run for prognosis and as a potential therapeutic target. *BAP1/ − 3p21* and *FBXW7/-chr4* are reported to be early clonal events, while *NF2*/ − 22q is considered late event [51]. Rare *ALK* (*STERN-ALK* most frequent) and *EWSR1* (*EWSR1-ATF1* or *EWSR1/FUS-CREB*) fusions have been observed in pleural mesothelioma of young patients. PD-L1 is highly expressed in SM and is associated with a poor prognosis [52, 53]. Indeed, testing for PD-L1 may be required in certain settings but not as a routine test. In some countries, immunotherapeutic agents are approved by the regulatory agencies for use in sarcomatoid mesothelioma, but for epithelioid mesothelioma they are only used if a PD-L1 expression of at least 1% is observed. Finally, the MESOMICS project reported a novel morpho-molecular classification of pleural mesothelioma based on four dimensions: ploidy, tumour cell morphology, adaptive immune response, and CpG island methylator profile, all having prognostic value and with potential therapeutic targets [54].

#### Lung carcinoma

The first cases relating asbestos exposure to lung carcinoma were reported in the 1930s [55], and until the 1970s, there was not a well-established connection between the different asbestos fibres and lung carcinoma [56–58]*.* Although the relative risk of developing lung carcinoma is significantly lower compared to the risk of mesothelioma development, still around 4% of lung carcinoma can be attributed to asbestos exposure [59]*.* It is also known that smoking in asbestos-exposed populations has a multiplicative rather than additive effect (× 80) [60]***.*** Asbestos fibres enable a higher uptake of cigarette smoke carcinogens in lung epithelial cells [61]***.*** Cigarette smoke also strengthens the binding of asbestos fibres to the same cells [62]***.*** This interaction results in genetic damage and finally malignant transformation of those cells.

Today, there is no clinical, radiological, or pathological way to discriminate asbestos-related carcinoma from asbestos-unrelated carcinoma [63]. Even studies about molecular profiling of the tumour are not straightforward in this distinction. The most controversial issue is whether the increased risk of lung carcinoma development is related to asbestos exposure per se or to asbestosis [8, 29, 64]. Advocates of the asbestosis hypothesis emphasize the potential of severe interstitial lung fibrosis to contribute to carcinogenesis, with carcinomas originating within areas of alveolar epithelial hyperplasia and with dysplasia occurring within fibrosis. Notably, these carcinomas tend to manifest more frequently within lung regions particularly affected by severe asbestosis, such as the lower lobes and the lung periphery. In contrast, proponents of the asbestos exposure hypothesis position it as both an initiator and promoter of carcinoma development, regarding fibrogenesis and carcinogenesis as separate outcomes. Supporting this hypothesis is also the fact that a significant proportion of carcinomas arising in the context of asbestos exposure are observed to emerge primarily within the bronchi, rather than within the alveolar tissue. Recent statistical data supports this hypothesis as well [63].

Subsequently, various countries have established distinct regulations and requirements for attributing lung carcinoma to asbestos exposure.

## Lung diseases caused by toxic fumes and gases

Exposure to vapours, fumes, and other airborne pollutants in occupational settings poses significant health risks, often leading to various respiratory ailments and systemic disorders. For instance, workers in industrial facilities where welding occurs are frequently exposed to welding fumes, which contain a complex mixture of metal oxides and gases. Prolonged inhalation of these fumes can result in respiratory conditions such as bronchitis, pneumonia, and even metal fume fever, characterized by flu-like symptoms such as fever, chills, and cough. Similarly, individuals working with chemical solvents, such as those found in paint thinners or industrial cleaning agents, may experience adverse respiratory effects due to exposure to volatile organic compounds. Chronic inhalation of these compounds has been linked to conditions like asthma, chronic bronchitis, and lung cancer. Moreover, firefighters regularly encounter hazardous smoke and toxic gases during firefighting operations, which can lead to acute respiratory distress and long-term lung damage. Inhalation of combustion byproducts like carbon monoxide, hydrogen cyanide, and polycyclic aromatic hydrocarbons can cause asphyxiation, chemical pneumonitis, and increase the risk of developing respiratory cancers [65].

Exposure to gases, vapours, and fumes in the workplace has the potential to cause almost any major type of lung disease in susceptible individuals. These triggers are frequently overlooked, as there is no rigid differentiation between general and occupational pollution. Inhaled substances may affect the respiratory system at various levels according to diverse factors (e.g., characteristics of substances, environmental and host factors, absorption into systemic circulation). In particular, highly water-soluble gases and vapours and larger mist or dust particles (greater than 10 µm in diameter) are generally deposited in the upper airways, while less soluble gases, vapours, and smaller particles can be inhaled more deeply into the respiratory tract [66]. Chemical irritants, asphyxiants, toxic metals, products of fires and combustion, and many other substances have been reported to cause lung injury mainly at the airway tract with features of different grades of inflammation [66]**.** In addition, a wide variety of chronic pulmonary complications may occur, even if in less than 10% of all exposed individuals. Among these, the most frequent is the reactive airway disease syndrome (RADS), which is a form of occupational asthma characterized by a sudden onset and persistence of airway reactivity that may develop in individuals who are acutely exposed to high concentrations of an irritant product. Another chronic affection is bronchiolitis obliterans (± organizing pneumonia), also known as obliterative bronchiolitis or constrictive bronchiolitis and reported to be most frequently associated with exposure to butter flavour chemical products used in popcorn production, oxides of nitrogen, sulphur dioxide, chlorine, ammonia, and phosgene [67]. Finally, COPD, bronchiectasis, diffuse alveolar damage (DAD), lung fibrosis, and lung cancer can also be aetiologically correlated to occupational exposure to toxic fumes and gases, but the cause-effect relation is not always easy to demonstrate, and pathologic features do not show pathognomonic aspects that can help in the diagnosis.

### Key morphological features

Mainly airway remodelling, characterized by chronic inflammation, increased mucus production and narrowing of the airways contributing to respiratory diseases such as COPD and occupational asthma.

### Supportive analyses

- *Prussian blue stain*: In cases where toxic exposure involves inhalation of iron-containing particles (e.g., welding fumes); this staining can aid in identifying hemosiderin-laden macrophages.
- *Toluidine blue stain*: Inflammatory responses involving mast cell activation can be visualized, providing insights into the immune response to toxic exposures.

## Organic agents

Organic agent-induced pulmonary diseases, also known as organic dust diseases, encompass respiratory conditions caused by inhaling organic materials and allergenic substances. Common causative agents include bird proteins from feathers and droppings, mould spores due to water damage, and agricultural materials such as mouldy hay. Additionally, occupational exposures to chemicals like isocyanates and metalworking fluids can elicit HP, often in combination with other sensitizing agents. Over time, certain exposures have become less prevalent, while others have emerged, reflecting changes in workplace practices and recreational activities. Although the exact duration and intensity of exposure required to induce HP remain unclear, persistent and intense exposure is likely to contribute to disease progression. Some current occupation exposures are reported in Table 10*.*

**Table 10:** Occupation exposure profiles of organic agents

Occupation	Exposure
*Agricultural workers*	Organic dust from crops, mold, pesticides
*Woodworkers*	| wood dust
*Bakers and flour mill workers*	Flour dust
*Chemical industry workers*	Organic solvents, vapours, dust
*Healthcare workers*	Bioaerosols, disinfectants, pharmaceuticals
*Welders*	Welding fumes
*Painters*	Organic solvents in paints and coatings
*Garage workers/auto mechanics*	Organic solvents, exhaust fumes, dust
*Textile industry workers*	Organic dust, fibres

A specific type of organic agent-induced pulmonary diseases is HP, characterized by an exaggerated immune response to inhaled organic antigens leading to lymphocytic inflammation, granuloma formation, and potential fibrosis in the lung tissue. Upon inhalation, antigen-presenting cells, including macrophages and dendritic cells, interact with antigens via pattern recognition receptors, initiating a Th1 immune response. Concurrently, B cells produce IgG antibodies, activating the complement cascade and stimulating macrophages. Pro-inflammatory cytokines and chemotactic factors released by activated macrophages further enhance the immune response, leading to lymphocytic infiltration and granuloma formation. The transition from acute inflammation to chronic fibrosis involves a shift towards a Th2 immune response, inhibition of regulatory T cells, and upregulation of NKT cells. Genetic variants within the major histocompatibility complex and telomere-related genes, as well as respiratory tract infections and pesticide exposure, contribute to individual susceptibility to HP [68].

HP has a variable incidence and prevalence, influenced by factors like diagnostic criteria, geographical variations in antigen exposure, and host factors. Swedish farmers experience an incidence of around 20 per 100,000 person-years, while European registries report an incidence equal to 1.5 to 12% of all interstitial lung diseases (https://www.ncbi.nlm.nih.gov/books/NBK499918/). As in other occupational thoracic/lung diseases, a multidisciplinary approach is mandatory. The new guidelines from the American Thoracic Society (ATS) and the American College of Chest Physicians (ACCP) recommend a more straightforward approach: categorizing HP as “nonfibrotic” and “fibrotic” forms with different level of diagnostic certainty (definite, probable, and indeterminate) [69]. The histopathologic characteristics of HP are typically those of a chronic inflammatory interstitial pneumonia with bronchiolocentricity and small, indistinct non-necrotizing granulomas and/or multinucleated giant cells sometimes with refractile oxalate crystals or cholesterin crystal clefts. In the chronic form, dense fibrosis with microscopic honeycombing and collagen bridging formations between peribronchial areas and subpleural scarring may be seen. The histopathology of HP raises several differential diagnoses including nonspecific interstitial pneumonia (NSIP) for non-fibrotic HP and UIP/idiopathic pulmonary fibrosis (IPF) for fibrotic HP.

**Fig. 7:**
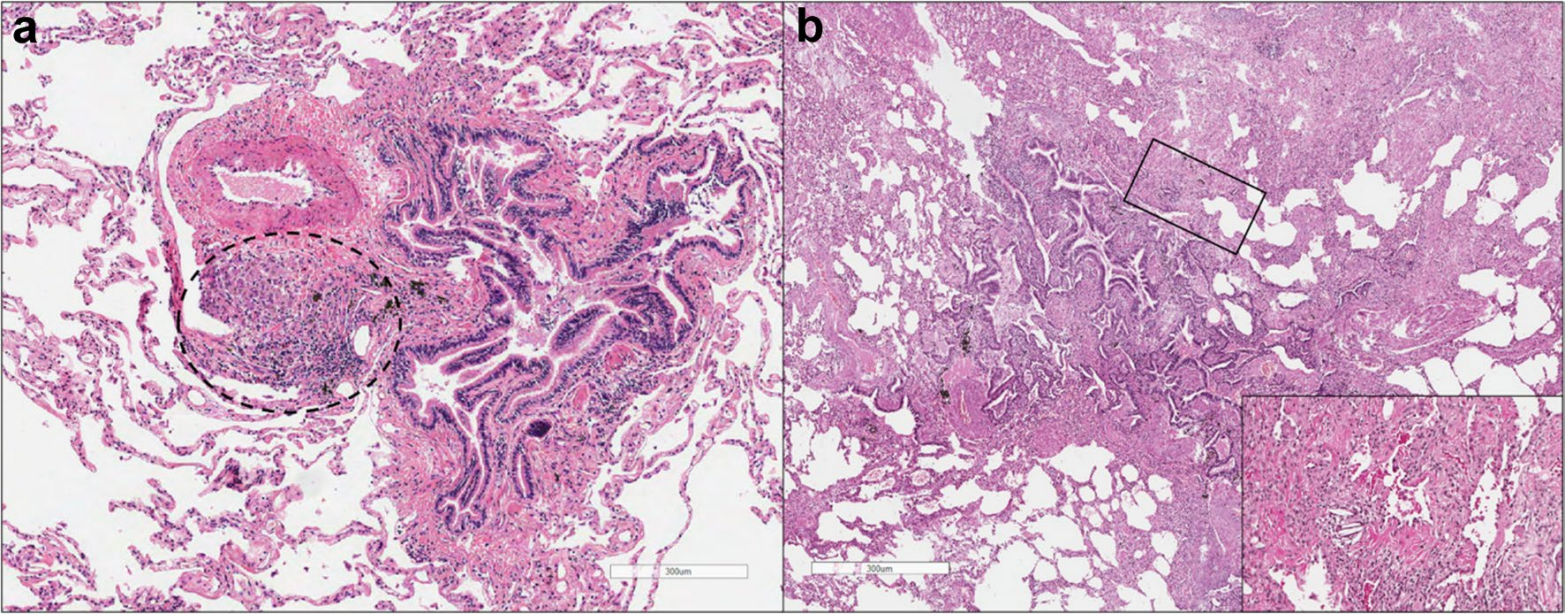
Histological features of non-fibrotic and fibrosing hypersensitivity pneumonitis (HP). **a** In non-fibrotic HP, mild to moderate inflammatory infiltrates, lymphocytes, and small granulomas (black dotted circle) within the lung parenchyma are depicted. **b** Contrastingly, in fibrosing HP, the histological examination highlights the presence of fibrosis within the lung interstitium, and overall the bronchiolocentric architectural distortion with peribronchial metaplasia and abortive granulomas with giant cells containing cholesterol clefts (**a, b**: haematoxylin and eosin staining, scale bar: 300 µm. Inset in **b** showing macrophages with cholesterol clefts)

Figure 7a, b shows the histological features of non-fibrotic and fibrotic HP.

### Key morphological features

- *Non-fibrotic HP*: a confident histopathological diagnosis requires the presence of typical features:
  - bronchiolocentric interstitial pneumonia.
  - cellular chronic bronchiolitis.
  - distinctive granulomatous inflammation.
  - the absence of histopathological features suggesting alternative diagnoses.
  Interstitial pneumonia is lymphocyte-predominant, bronchiolocentric, and polymorphic, while chronic bronchiolitis involves peribronchiolar interstitium expansion by a lymphocyte-predominant inflammatory infiltrate. Granulomatous inflammation, typically small and poorly formed, completes the triad for a confident diagnosis, but the diagnostic value depends on qualitative features, with well-formed granulomas raising the likelihood of other conditions. The presence of peribronchiolar metaplasia (PBM) or organizing pneumonia may be observed.
- *Fibrotic HP*: chronic interstitial pneumonia and bronchiolitis + fibrosis (subpleural and centriacinar fibrosis) sometimes with bridging fibrosis. Centriacinar fibrotic lesions, along with other characteristic features, serve as clues for a more likely diagnosis of fibrotic HP. The presence of fibrosis should be documented for prognostic considerations.

### Supportive analyses

- *BAL cell count*: increased lymphocytosis (> 30%) is highly suggestive even if these features lack a validation in most recent international consensus/guidelines.
- *Special stains for microorganisms (Giemsa, Gomori-Grocott, and Ziehl–Neelsen)* aid evaluation for infectious disease.
- *Immunostaining for cathepsin and CD68* may be helpful to detect small granulomas.

## The industrial postmortem examination

In suspected deaths due to occupational disease, the role of the autopsy is (1) to describe and diagnose all occupational/industrial disease manifestations, (2) to determine disease causation, and (3) to determine and record concomitant disease present that may have impacted life expectancy distinct to the defined occupational disease. This will be taken into consideration when evaluating personal injury loss in legal claims. The most encountered toxin-related fatal diseases are due to cancer (mesothelioma and/or lung cancer) or lung fibrosis (pneumoconiosis).

### Clinical information

The circumstances of death are important so there should be full access to the medical records. This is important when determining disease causation and any impact of known confounding risk factors, such as smoking in lung cancer cases, and collagen vascular disease in interstitial lung disease.

### The exposure history

Toxic mineral dust exposures may be encountered in the workplace (occupational setting—direct or indirect bystander type), from domestic exposures or “take-home” paraoccupational sources or from environmental sources. There should be a careful consideration of all these factors when assessing disease and disease causation. Patterns of exposure are important—almost everyone has mixed low-level exposures to a wide variety of minerals including asbestos, silica, and silicates from ambient air breathing—none of these background exposures result in any increased risk of disease. Occupational lung diseases are associated with cumulative exposures which are orders of magnitude above background ambient levels of mineral dusts.

The diagnosis of coal workers pneumoconiosis, silicosis, silicatosis, and asbestos-related diseases—mesothelioma, lung cancer, and lung fibrosis/asbestosis—is set out above.

This section provides a useful guide for determining disease causation.

### Asbestos-related disease

At postmortem for confirmed cases of pleural mesothelioma, asbestos causation may reasonably be concluded when: there are associated biomarkers of exposure—asbestosis, pleural plaques, or identified asbestos bodies by light microscopy; these correlate closely with above background exposures to amphibole asbestos, or when exposure history is commensurate with the development of the disease.

For subjects with either confirmed lung cancer or lung fibrosis, asbestos causation (as asbestos-related lung cancer or asbestosis respectively) is concluded when there is either concomitant criteria fulfilling the CAP-PPS asbestosis committee [38], or a fibre count within the asbestosis range for the analytical laboratory. Light microscopic mineral analytical methods are not advocated as they provide no qualitative information and are insensitive. Electron microscopic mineral analysis allows for an accurate determination of retained minerals, especially amphibole asbestos fibres sufficient to induce disease. Such analytical methods can determine asbestiform versus non-asbestiform cleavage fragments, which are biologically inert. For predominantly chrysotile asbestos exposures, the occupational history may yield additional information. It is necessary for analytical laboratories to establish control reference ranges for subjects with no disease, as well as those with asbestosis. For subjects with mesothelioma and no clear exposure history, or biomarkers of asbestos exposure, disease is likely unrelated to asbestos either due to other mineral fibres, radiation, inherited genetic factors, or acquired mutations with age, as naturally occurring cancers/spontaneous cancer. Presently, most men with pleural mesothelioma have disease due to prior amphibole asbestos exposures; for women, paraoccupational or environmental exposures are proportionately more relevant as is naturally occurring cancer. Nowadays, most peritoneal mesotheliomas arise unrelated to asbestos as genetic diseases.

### Silica, silicates, and coal

Significant clinical disease and mortality are associated with the manifestation of either complicated pneumoconiosis, progressive massive fibrosis, or complications of emphysema such as pneumonia respiratory failure or cor pulmonale.

### Unusual pathology with uncertain exposure

In certain settings the pathological picture provides an overlapping mixture of histological changes which may originate from different sources and exposures. In this setting, an electron microscopic fibrous and non-fibrous mineral analysis is useful to determine the diagnosis.

### Autopsy summary

The pathologist should provide a clear clinicopathological summary outlining the gross and histological findings of the disease and its causation, incorporating any mineral analytical data as well as any significant unrelated disease conditions.

## Multidisciplinary approach

### The contribution of occupational specialists

The occupational physician is the expert in characterizing occupational exposure and linking it with adverse health effects.

Several tools are available that can provide estimates or measurements of exposure(s).

The *first* tool, as Ramazzini taught us [70], is the documentations of an effective occupational history, which requires time and experience, but is the basis of an accurate diagnosis. There are some ways to promote this process: (1) asking the patient to fill in a chronological occupational history template, which reports data on current and former duties and can be discussed during the consultation with the occupational physician; and then (2) surveying the patient about chemicals and dust-control measures (e.g., use of respiratory protective equipment, wet processing, and dust extraction). When taking the occupational history, the occupational physician can link specific exposures with specific target organs or systems and always considers that (a) the effect of many agents requires a (long) latency to appear; (b) in the occupational settings, many different hazards can be encountered simultaneously and may have a synergistic effect. In addition, it is relevant to ask about the health of co-workers who might have developed symptoms similar to those of the patient.

The occupational physician has also the expertise in using the information reported in the safety data sheets of the products, which represent, whenever available, a *second* precious tool to characterize the exposure, including that to respiratory hazards.

A *third* useful tool isto collect the data provided by the industrial hygienists, i.e., derived from personal or static measurements of airborne pollutants performed at the workplace. If these—preferred—objective measurements are not feasible or are too expensive, the occupational physician can derive information from estimates of exposure reported in databases of national health and safety authorities or in the literature.

The occupational physician, as an expert in safety and health surveillance who can also visit workplaces, matches all the information collected from the beforementioned activities and contributes to making the most accurate diagnosis possible of lung diseases, avoiding the diagnostic odyssey that many patients suffer from.

### The contribution of pulmonologists

Pulmonologists when dealing with interstitial lung disease have the major challenge of assessing the occupational burden both in the idiopathic forms (i.e., idiopathic interstitial pneumonias, IIPs) and the non-idiopathic forms such as fibrosing HP or the classic pneumoconiosis (e.g., asbestosis, silicosis). Indeed, a recent American Thoracic Society (ATS) statement highlighted that domestic or work exposures may contribute to the burden of a disease otherwise considered idiopathic (i.e., patients with IPF in 26% of cases) [71]*.* Idiopathic and occupational ILDs share some common structural abnormalities and thus can look similar from a functional viewpoint. On the other hand, ILDs due to inhaled exposures have a better outcome in patients that avoid the causing agents. Nevertheless, it is essential that an expert pulmonologist in the field of ILD obtains a complete and detailed history about any associated exposure to biological products, gases, chemicals, vapours, dust, and/or fumes ascertaining the intensity and duration of the exposure as much as possible.

Key to this complex diagnostic process would be the use of a standardized questionnaire to minimize misclassification and/or the risk of overestimating occupational exposure. Although such objective questionnaires are being increasingly developed, no internationally standardized template has yet been accepted [72]*.* It is still a difficult task to establish a direct causative role of environmental and/or occupational exposures which, before anything else, requires a multidisciplinary discussion (MDD). Indeed, even though a MDD has been recognized as the gold standard for ILD diagnosis [73], different expert multidisciplinary teams have previously reported unsatisfactory agreement, in particular when dealing with the suspicion of hypersensitivity pneumonitis for which it is difficult and crucial to identify the role of environmental and domestic exposure as a possible inciting agent [74]. Therefore, because of the complexity of the occupational interview, an occupational physician should participate in the multidisciplinary team and help collecting all crucial information, at least on a case-by-case basis [71]**.** This is also suggested by an official ATS/European Respiratory Society (ERS) joint report which identified the urgent need to improve knowledge about the role of occupational factors in the context of non-malignant respiratory diseases***.*** A recent study conducted on patients with ILD, which reviewed the cases during a MDD and offered a consultation with an occupational physician, showed a high prevalence of occupational exposure; indeed, about two thirds of the patients with ILD had some respiratory exposure: in 41 out of 141 patients, the hypothesis of occupational origin was plausible, and for 15 of them, an occupational disease compensation procedure was initiated following an occupational disease consultation [75]. Finally, legal and financial implications for individual patients reinforce the added value of including an occupational physician in the ILD multidisciplinary discussions.

### The contribution of thoracic radiologists

Imaging plays a central role in the screening and diagnosis of pneumoconiosis as well as in the differential diagnosis and in the diagnosis of complications. For decades, chest radiographs have been the imaging modality of choice in patients with suspected or known pneumoconiosis. To unify the classification of chest radiograph findings, the International Labor Organization (ILO) published a classification system which is used worldwide (https://www.ilo.org/wcmsp5/groups/public/---ed_dialogue/---lab_admin/documents/publicationwcms_867859.pdf) [78]. The main advantages of chest radiographs are their wide availability, relatively low costs, and low radiation dose. These radiographs are thus indicated to screen for pneumoconiosis as well as for initial imaging. While chest radiographs will remain the main imaging modality in countries with limited resources, thin section computed tomography (CT) without contrast is being used more and more for the initial imaging and for further evaluation [76]***.*** Already in 2005, Kusaka et al. highlighted the potential role of high-resolution (HR) CT for this group of diseases [77]***.*** For example, CT has been shown to be crucial in identifying and characterizing accelerated silicosis in Turkish denim sandblasters [78]*.* Thus, CT assists clinicians and pathologists in verifying suspected diagnoses and may also help identify new exposures [77]**.** Occupational lung diseases can be classified, according to the predominant CT pattern, as diffuse lung opacities (ground-glass, crazy paving, and consolidations), nodules, reticular abnormalities, masses, cysts, and emphysema [31]*.* The differential diagnosis is based primarily on the predominant CT pattern and its distribution. For example, silicosis and coal worker pneumoconiosis are characterized by perilymphatic nodules, whereas centrilobular nodules are typically seen in siderosis, hypersensitivity pneumonitis, acute silicosis, and hard metal lung disease [79, 80]***.*** Given the importance of imaging in the diagnosis of pneumoconiosis, machine learning and artificial intelligence algorithms have also been increasingly used in this field in recent years*.* For example, Zhang et al. achieved promising results with a deep learning-based model for screening and grading pneumoconiosis from chest radiographs, outperforming two groups of radiologists [81–83]. Despite these encouraging results, some challenges still need to be overcome to translate such tools into clinical practice, as imaging in occupational lung disease is very heterogeneous and further models need to be developed based on CT and possibly including multidisciplinary data from occupational exposure, clinical assessment, histology, and radiology.

## What pathologists should seek to advance knowledge: the view of occupational/pulmonologist specialists

The collaboration between pathology and occupational medicine holds great potential for advancing our understanding of occupational diseases and enhancing patient care.

One of the most pressing issues concerns the emergence of cleaning-related respiratory diseases and silicosis due to exposure to “artificial stone,” which poses a significant challenge for occupational physicians [84].

Work exposure to cleaning products is increasingly common and can lead to work-related asthma (by sensitization, irritant induced, or work-exacerbated) or COPD. The inflammatory pathways underlying these phenotypes are still unknown.

In order to identify the distinct endotypes associated with various disease phenotypes, there is a need for innovative methods to uncover immunopathological traits and genetic markers. These approaches are crucial for guiding health surveillance initiatives in cleaning sectors.

It is necessary to implement the knowledge on the pathological pattern of the artificial stone silicosis. A comprehensive analysis of the silicotic nodules could elucidate whether other noxious agents, in addition to crystalline silica, may act as inflammation and fibrosis triggers, i.e., resins and metals (Al, Na, Fe, Ca, Ti) [85]**.** Synthetic mineral fibres, including slag wool, glass wool, rockwool, glass filaments, microfibres, and refractory ceramic fibres, exhibit toxicity influenced by various factors such as diameter, length, biopersistence, physicochemical structure, surface properties, and exposure level. While their chemical composition depends on the raw material, most are amorphous silicates combined with metal oxides and additives.

Considering the frequent clinical conundrums in the differential diagnosis between silicosis and sarcoidosis, a careful occupational history and a proper mineralogic analysis of lung tissue is important to provide a timely diagnosis and appropriate management of the disease [86]**.**

Sarcoidosis continues to be considered an idiopathic disease, but it is open to question whether this is true. Recent case reports and epidemiologic studies have revealed significant associations between occupational exposures (silica included) and this disease. However, more detailed investigations are needed [87]**.**

Another important topic that pathologists should actively investigate is the diagnosis of early forms of mesothelioma (mesothelioma in situ) and explore new treatment approaches, particularly in the field of immunotherapy. The early detection of mesothelioma is crucial to improve patient outcomes, and pathologists can play a pivotal role in developing diagnostic criteria and techniques for identifying these initial disease stages and their propensity for progression into full-blown invasive disease. Additionally, it is essential to understand the intricate histopathological features of mesothelioma and their potential influence on immunotherapy responsiveness.

## Final pathological report

The final pathological report should include the histomorphological pattern of lung pathology, e.g., diffuse alveolar damage or fibrosis, and the presence or absence of specific characteristics pointing towards an occupational lung disease, e.g., asbestos bodies or silico-anthracotic nodules. Most pigmented peribronchiolar histiocytic lesions will be not specifically summarized as mixed-dust nodules. The final diagnosis of an occupational disease is an interdisciplinary endeavour, and not just the subject of a pathology report [88]. Standardizing language in pulmonary occupational lung diseases, akin to practices in oncology, is crucial for accurate diagnoses, effective communication, and comprehensive patient care. This precision is vital for informed decision-making by healthcare professionals, contributing to improved patient management. Standardized reporting is also strongly encouraged for reporting neoplastic diseases, including lung cancer and mesothelioma, and for more completely reporting pathological results. Constantly updated templates, consisting of both obligatory core elements and facultative non-core elements, are available from the ICCR project (improving outcomes for cancer patients by standardizing pathology reporting) [89] and national societies, such as the College of American Pathologists (CAP) and the Royal Collage of Pathologists (RCPath) [90]***.*** Regarding findings suggesting occupational lung diseases, the presence or absence of asbestos bodies or pleural plaques is specifically listed under “additional findings” in the pleural mesothelioma CAP cancer protocol, the ICCR templates [89, 91], or the RCpath [90]—data minimum-sets. Contrary to the USA, where standardized reporting using the CAP templates is mandatory for accredited Institutes of Pathology, in Europe it is only recommended and may be adapted on a voluntary basis [92].

The importance of MDT discussions, well-regulated in the management of interstitial lung diseases, should also be emphasized for occupational diseases. We have highlighted the need for regulatory statements to define who should participate in MDT meetings, how they should be conducted, and the importance of standardized reporting. Such standardization facilitates data convergence for research and epidemiological surveillance, enhancing our understanding of disease trends.

Standardized language facilitates data convergence for research and epidemiological surveillance, enhancing our understanding of disease trends. Consistent terminology in education ensures a shared understanding among medical professionals, and compliance with regulations is streamlined. Patient empowerment is heightened through clear communication, and standardization fosters global collaboration and information sharing, promoting research initiatives and best practices. In adopting language standardization from oncology, the field of occupational respiratory health stands to benefit significantly.

## Limitations of the study

This manuscript has some limitations. It primarily serves as a descriptive educational resource rather than presenting new findings. The focus on certain occupational lung diseases provides morphological descriptions but lacks comprehensive data or hypotheses on their pathogenesis. Additionally, specific discussions on disease mechanisms are missing.

However, we acknowledge that our primary aim in this review was the morphological presentation of these pneumoconioses. We intentionally omitted detailed discussions on pathophysiological mechanisms due to space constraints, aiming to meet our primary goal of addressing the needs of pathologists. In our efforts to maintain brevity, we aimed to provide a focused and visually informative resource tailored to their needs.

Recognizing these shortcomings, we understand the need for a more detailed and comprehensive review. Future iterations of this manuscript will aim to incorporate more in-depth discussions on the pathogenesis of occupational lung diseases, the specific mechanisms of metal toxicity, the health impacts of air pollution, and a thorough examination of man-made mineral fibres and their associated toxicities. Additionally, we will strive to provide a balanced review that integrates both descriptive content and current research findings, offering a more robust and informative resource.

## References

[1] Vlahovich KP, Sood A (2021) A 2019 Update on occupational lung diseases: a narrative review. Pulm Ther 7:75–87. 10.1007/s41030-020-00143-4 33385174 10.1007/s41030-020-00143-4 PMC8137769

[2] De Matteis S, Heederik D, Burdorf A et al (2017) Current and new challenges in occupational lung diseases. Eur Respir Rev an Off J Eur Respir Soc 26:. 10.1183/16000617.0080-2017 10.1183/16000617.0080-2017 PMC6033059 29141963

[3] Tarlo SM (2012) Occupational lung disease. Goldman's Cecil Med 567–574. 10.1016/B978-1-4377-1604-7.00093-2

[4] Corrin B, Nicholson AG (2011) Occupational, environmental and iatrogenic lung disease. Pathol Lungs 327–399. 10.1016/B978-0-7020-3369-8.00007-0

[5] Goodwin RA, Des Prez RM (1983) Apical localization of pulmonary tuberculosis, chronic pulmonary histoplasmosis, and progressive massive fibrosis of the lung. Chest 83:801–805. 10.1378/chest.83.5.801 6839825 10.1378/chest.83.5.801

[6] Bake B, Wood L, Murphy B et al (1974) Effect of inspiratory flow rate on regional distribution of inspired gas. J Appl Physiol 37:8–17. 10.1152/jappl.1974.37.1.8 4836560 10.1152/jappl.1974.37.1.8

[7] Davidson KR, Ha DM, Schwarz MI, Chan ED (2020) Bronchoalveolar lavage as a diagnostic procedure: a review of known cellular and molecular findings in various lung diseases. J Thorac Dis 12(9):4991–5019. 10.21037/jtd-20-651 10.21037/jtd-20-651 PMC7578496 33145073

[8] Sporn TRV (2013) Spencer’s pathology of the lung. In: Hasleton P, Flieder DB (eds), 6th edn. Cambridge University Press

[9] Shen F, Sergi C (2023) Sputum analysis. In: StatPearls. StatPearls Publishing 33085342

[10] Calabrese F, Lunardi F, Pezzuto F et al (2019) Are there new biomarkers in tissue and liquid biopsies for the early detection of non-small cell lung cancer? J Clin Med 8:1–20. 10.3390/jcm8030414 10.3390/jcm8030414 PMC6463117 30917582

[11] Pezzuto F, Fortarezza F, Lunardi F, Calabrese, F (2019) Are there any theranostic biomarkers in small cell lung carcinoma? J Thorac Dis 11(Suppl 1):S102–S112. 10.21037/jtd.2018.12.14 10.21037/jtd.2018.12.14 PMC6353747 30775033

[12] Jary H, Rylance J, Patel L et al (2015) Comparison of methods for the analysis of airway macrophage particulate load from induced sputum, a potential biomarker of air pollution exposure. BMC Pulm Med 15:137. 10.1186/s12890-015-0135-7 26542371 10.1186/s12890-015-0135-7 PMC4635991

[13] Kodros JK, O’Dell K, Samet JM et al (2021) Quantifying the health benefits of face masks and respirators to mitigate exposure to severe air pollution. GeoHealth 5:e2021GH000482. 10.1029/2021GH000482 34541439 10.1029/2021GH000482 PMC8438762

[14] de Assis MP, Barcella RC, Padilha JC et al (2021) Health problems in agricultural workers occupationally exposed to pesticides. Rev Bras Med do Trab publicacao Of da Assoc Nac Med do Trab 18:352–363. 10.47626/1679-4435-2020-532 10.47626/1679-4435-2020-532 PMC7879472 33597986

[15] Hamzah NA, Mohd Tamrin SB, Ismail NH (2016) Metal dust exposure and lung function deterioration among steel workers: an exposure-response relationship. Int J Occup Environ Health 22:224–232. 10.1080/10773525.2016.1207040 27392157 10.1080/10773525.2016.1207040 PMC5102237

[16] Maiti KS (2023) Non-invasive disease specific biomarker detection using infrared spectroscopy: a review. Molecules 28:. 10.3390/molecules28052320 10.3390/molecules28052320 PMC10005715 36903576

[17] López-Cima MF, Alvarez-Avellón SM, Pascual T et al (2012) Genetic polymorphisms in CYP1A1, GSTM1, GSTP1 and GSTT1 metabolic genes and risk of lung cancer in Asturias. BMC Cancer 12:433. 10.1186/1471-2407-12-433 23013535 10.1186/1471-2407-12-433 PMC3518149

[18] Nishida C, Yatera K (2022) The impact of ambient environmental and occupational pollution on respiratory diseases. Int J Environ Res Public Health 19. 10.3390/ijerph19052788 10.3390/ijerph19052788 PMC8910713 35270479

[19] Gonzalez-Covarrubias V, Martínez-Martínez E, Del Bosque-Plata L (2022) The potential of metabolomics in biomedical applications. Metabolites 12:. 10.3390/metabo12020194 10.3390/metabo12020194 PMC8880031 35208267

[20] Gao R, Wang F, Wang Z et al (2019) Diagnostic value of soluble mesothelin-related peptides in pleural effusion for malignant pleural mesothelioma: an updated meta-analysis. Medicine (Baltimore) 98:e14979. 10.1097/MD.0000000000014979 30946324 10.1097/MD.0000000000014979 PMC6456135

[21] Sorino C, Mondoni M, Marchetti G et al (2023) Pleural mesothelioma: advances in blood and pleural biomarkers. J Clin Med 12:. 10.3390/jcm12227006 10.3390/jcm12227006 PMC10672377 38002620

[22] Katzenstein AA, Askin FB (1982) Surgical pathology of non-neoplastic lung disease. Major Probl Pathol 13:1–430 7087547

[23] Roberts WC (2002) Pulmonary talc granulomas, pulmonary fibrosis, and pulmonary hypertension resulting from intravenous injection of talc-containing drugs intended for oral use. Proc (Bayl Univ Med Cent) 15:260–261. 10.1080/08998280.2002.11927851 16333448 10.1080/08998280.2002.11927851 PMC1276621

[24] Leonard R, Zulfikar R, Stansbury R (2020) Coal mining and lung disease in the 21st century. Curr Opin Pulm Med 26:135–141. 10.1097/MCP.0000000000000653 31815751 10.1097/MCP.0000000000000653

[25] Graf BA, Kirk J Van, Sime PJ (2023) Fibroblasts: implications for pulmonary fibrosis.10.1152/ajplung.00024.2004.Crystalline

[26] Leung CC, Yu ITS, Chen W (2012) Silicosis. Lancet (London, England) 379:2008–2018. 10.1016/S0140-6736(12)60235-9 22534002 10.1016/S0140-6736(12)60235-9

[27] Barnes H, Goh NSL, Leong TL, Hoy R (2019) Silica-associated lung disease: an old-world exposure in modern industries. Respirology 24:1165–1175. 10.1111/resp.13695 31517432 10.1111/resp.13695

[28] Silicosis and Silicate Disease Committee (1988) Diseases associated with exposure to silica and nonfibrous silicate minerals. Arch Pathol Lab Med 112(7):673–720 2838005

[29] Gibbs A, Attanoos RL, Churg A, Weill H (2007) The “Helsinki criteria” for attribution of lung cancer to asbestos exposure: how robust are the criteria? Arch Pathol Lab Med 131:181–183 17284100 10.5858/2007-131-181-THCFAO

[30] Antao VC dos S, Pinheiro GA, Terra-Filho M et al (2005) High-resolution CT in silicosis: correlation with radiographic findings and functional impairment. J Comput Assist Tomogr 29:350–356.10.1097/01.rct.0000160424.56261.bc 10.1097/01.rct.0000160424.56261.bc 15891506

[31] Cox CW, Rose CS, Lynch DA (2014) State of the art: imaging of occupational lung disease. Radiology 270:681–696. 10.1148/radiol.13121415 24568704 10.1148/radiol.13121415

[32] Vanka KS, Shukla S, Gomez HM et al (2022) Understanding the pathogenesis of occupational coal and silica dust-associated lung disease. Eur Respir Rev 31:. 10.1183/16000617.0250-2021 10.1183/16000617.0250-2021 PMC9724915 35831008

[33] Roggli VL (1994) Rare pneumoconiosis: metalloconioses. In: Mj S (ed) Pathology of pulmonary disease. Lippincott, Philadelphia, pp 411–422

[34] De Vuyst P, Dumortier P, Schandené L et al (1987) Sarcoidlike lung granulomatosis induced by aluminum dusts. Am Rev Respir Dis 135:493–497. 10.1164/arrd.1987.135.2.493 3813209 10.1164/arrd.1987.135.2.493

[35] Balmes JR, Abraham JL, Dweik RA et al (2014) An official American Thoracic Society statement: diagnosis and management of beryllium sensitivity and chronic beryllium disease. Am J Respir Crit Care Med 190:e34-59. 10.1164/rccm.201409-1722ST 25398119 10.1164/rccm.201409-1722ST

[36] Naqvi AH, Hunt A, Burnett BR, Abraham JL (2008) Pathologic spectrum and lung dust burden in giant cell interstitial pneumonia (hard metal disease/cobalt pneumonitis): review of 100 cases. Arch Environ Occup Health 63:51–70. 10.3200/AEOH.63.2.51-70 18628077 10.3200/AEOH.63.2.51-70

[37] Kuroda A (2021) Recent progress and perspectives on the mechanisms underlying asbestos toxicity. Genes Environ 43:1–8. 10.1186/s41021-021-00215-0 34641979 10.1186/s41021-021-00215-0 PMC8507173

[38] Roggli VL, Gibbs AR, Attanoos R et al (2010) Pathology of asbestosis—an update of the diagnostic criteria: report of the Asbestosis Committee of the College of American Pathologists and Pulmonary Pathology Society. Arch Pathol Lab Med 134:462–480. 10.5858/134.3.462 20196674 10.5858/134.3.462

[39] Roggli V, Gibbs AR, Attanoos R et al (2016) Pathology of asbestosis: an update of the diagnostic criteria response to a critique. Arch Pathol Lab Med 140:950–952. 10.5858/arpa.2015-0503-SA 27575267 10.5858/arpa.2015-0503-SA

[40] Attanoos RL, Alchami FS, Pooley FD, Gibbs AR (2016) Usual interstitial pneumonia in asbestos-exposed cohorts—concurrent idiopathic pulmonary fibrosis or atypical asbestosis? Histopathology 69:492–498. 10.1111/his.12951 26864248 10.1111/his.12951

[41] Andujar P, Simon-Deckers A, Galateau-Sallé F et al (2014) Role of metal oxide nanoparticles in histopathological changes observed in the lung of welders. Part Fibre Toxicol 11:23. 10.1186/1743-8977-11-23 24885771 10.1186/1743-8977-11-23 PMC4037282

[42] Rice A, Nicholson AG (2009) The pathologist’s approach to small airways disease. Histopathology 54:117–133. 10.1111/j.1365-2559.2008.03175.x 19187181 10.1111/j.1365-2559.2008.03175.x

[43] Camiade E, Gramond C, Jutand M-A et al (2013) Characterization of a French series of female cases of mesothelioma. Am J Ind Med 56:1307–1316. 10.1002/ajim.22229 23939988 10.1002/ajim.22229

[44] Panou V, Røe OD (2020) Inherited genetic mutations and polymorphisms in malignant mesothelioma: a comprehensive review. Int J Mol Sci 21:. 10.3390/ijms21124327 10.3390/ijms21124327 PMC7352726 32560575

[45] WHO Classification of Tumours Editorial Board (2021) WHO classification of tumours series. In: WHO classification of tumours, 5th edn. International Agency for Research on Cancer, Lyon, France

[46] Galateau-Salle F, Hamilton T, MacNeill A et al (2023) Mesothelioma in situ mimicking well-differentiated papillary mesothelial tumor. Am J Surg Pathol 47:611–617. 10.1097/PAS.0000000000002033 36876759 10.1097/PAS.0000000000002033

[47] Beasley MB, Galateau-Salle F, Dacic S (2021) Pleural mesothelioma classification update. Virchows Arch 478:59–72. 10.1007/s00428-021-03031-7 33475835 10.1007/s00428-021-03031-7

[48] Churg A (2024) New developments in mesothelial pathology. Histopathology 84:136–152. 10.1111/his.15007 37694811 10.1111/his.15007

[49] Galateau Salle F, Le Stang N, Tirode F et al (2020) Comprehensive molecular and pathologic evaluation of transitional mesothelioma assisted by deep learning approach: a multi-institutional study of the international mesothelioma panel from the MESOPATH Reference Center. J Thorac Oncol Off Publ Int Assoc Study Lung Cancer 15:1037–1053. 10.1016/j.jtho.2020.01.025 10.1016/j.jtho.2020.01.025 PMC8864581 32165206

[50] Chapel DB, Schulte JJ, Husain AN, Krausz T (2020) Application of immunohistochemistry in diagnosis and management of malignant mesothelioma. Transl Lung Cancer Res 9:S3–S27. 10.21037/tlcr.2019.11.29 32206567 10.21037/tlcr.2019.11.29 PMC7082260

[51] Zhang M, Luo J-L, Sun Q et al (2021) Author correction: clonal architecture in mesothelioma is prognostic and shapes the tumour microenvironment. Nat Commun 12:3569 34099722 10.1038/s41467-021-23867-6 PMC8185116

[52] Pezzuto F, Lunardi F, Vedovelli L et al (2021) P14/ARF-positive malignant pleural mesothelioma: a phenotype with distinct immune microenvironment. Front Oncol 11:653497. 10.3389/fonc.2021.653497 33828993 10.3389/fonc.2021.653497 PMC8019896

[53] Pasello G, Zago G, Lunardi F et al (2018) Malignant pleural mesothelioma immune microenvironment and checkpoint expression: correlation with clinical-pathological features and intratumor heterogeneity over time. Ann Oncol Off J Eur Soc Med Oncol 29:1258–1265. 10.1093/annonc/mdy086 10.1093/annonc/mdy086 29514216

[54] Mangiante L, Alcala N, Sexton-Oates A et al (2023) Multiomic analysis of malignant pleural mesothelioma identifies molecular axes and specialized tumor profiles driving intertumor heterogeneity. Nat Genet 55:607–618. 10.1038/s41588-023-01321-1 36928603 10.1038/s41588-023-01321-1 PMC10101853

[55] Lynch K (1935) Two cases of squamous carcinoma of the lung occurring in asbestosis. Tubercle 17:5–10 10.1016/S0041-3879(35)80795-2

[56] Doll R (1955) Mortality from lung cancer in asbestos workers. Br J Ind Med 12(2):81–86. 10.1136/oem.12.2.81 10.1136/oem.12.2.81 PMC1037613 14363586

[57] Wagner JC, Sleggs CA, Marchand P (1960) Diffuse pleural mesothelioma and asbestos exposure in the North Western Cape Province. Br J Ind Med 17(4):260–271. 10.1136/oem.17.4.260 10.1136/oem.17.4.260 PMC1038078 13782506

[58] Selikoff IJ, Hammond ECCJ (1972) Carcinogenicity of amosite asbestos. Arch Env Heal 25:183–186 10.1080/00039896.1972.10666158 5048236

[59] Roggli VL, Oury TD, Sporn TA (2004) Carcinoma of the lung. In: Springer (ed) Pathology of asbestos-associated diseases, 2nd edn. New York, pp 193–216

[60] Olsson AC, Vermeulen R, Schüz J et al (2017) Exposure-response analyses of asbestos and lung cancer subtypes in a pooled analysis of case-control studies. Epidemiology 28:288–299. 10.1097/EDE.0000000000000604 28141674 10.1097/EDE.0000000000000604 PMC5287435

[61] Eastman A, Mossman BT, Bresnick E (1983) Influence of asbestos on the uptake of benzo(a)pyrene and DNA alkylation in hamster tracheal epithelial cells. Cancer Res 43:1251–1255 6297722

[62] Churg A (1996) The uptake of mineral particles by pulmonary epithelial cells. Am J Respir Crit Care Med 154:1124–1140. 10.1164/ajrccm.154.4.8887617 8887617 10.1164/ajrccm.154.4.8887617

[63] Klebe S, Leigh J, Henderson DW, Nurminen M (2019) Asbestos, smoking and lung cancer: an update. Int J Environ Res Public Health 17:. 10.3390/ijerph17010258 10.3390/ijerph17010258 PMC6982078 31905913

[64] Henderson DW, Rödelsperger K, Woitowitz H-J, Leigh J (2004) After Helsinki: a multidisciplinary review of the relationship between asbestos exposure and lung cancer, with emphasis on studies published during 1997–2004. Pathology 36:517–550. 10.1080/00313020400010955 15841689 10.1080/00313020400010955

[65] Manisalidis I, Stavropoulou E, Stavropoulos A, Bezirtzoglou E (2020) Environmental and health impacts of air pollution: a review. Front Public Heal 8:1–13. 10.3389/fpubh.2020.00014 10.3389/fpubh.2020.00014 PMC7044178 32154200

[66] Gorguner M, Akgun M (2010) Acute inhalation injury. Eurasian J Med 42:28–35. 10.5152/eajm.2010.09 25610115 10.5152/eajm.2010.09 PMC4261306

[67] Kreiss K, Gomaa A, Kullman G et al (2002) Clinical bronchiolitis obliterans in workers at a microwave-popcorn plant. N Engl J Med 347:330–338. 10.1056/NEJMoa020300 12151470 10.1056/NEJMoa020300

[68] Barnes H, Troy L, Lee CT et al (2022) Hypersensitivity pneumonitis: current concepts in pathogenesis, diagnosis, and treatment. Allergy Eur J Allergy Clin Immunol 77:442–453. 10.1111/all.15017 10.1111/all.15017 34293188

[69] Raghu G, Remy-Jardin M, Ryerson CJ et al (2020) Diagnosis of hypersensitivity pneumonitis in adults. An official ATS/JRS/ALAT Clinical Practice Guideline. Am J Respir Crit Care Med 202:e36–e69. 10.1164/rccm.202005-2032ST 32706311 10.1164/rccm.202005-2032ST PMC7397797

[70] Franco G, Franco F (2001) Bernardino Ramazzini: the father of occupational medicine. Am J Public Health 91:1382. 10.2105/AJPH.91.9.1382 11527763 10.2105/AJPH.91.9.1382 PMC1446786

[71] Blanc PD, Annesi-Maesano I, Balmes JR et al (2019) The occupational burden of nonmalignant respiratory diseases. An official American thoracic society and European respiratory society statement. Am J Respir Crit Care Med 199:1312–1334. 10.1164/rccm.201904-0717ST 31149852 10.1164/rccm.201904-0717ST PMC6543721

[72] Polke M, Kirsten D, Teucher B et al (2020) A comparison of existing questionnaires for identifying the causes of interstitial and rare lung diseases. Respiration 99:119–124. 10.1159/000504677 32000164 10.1159/000504677

[73] Raghu G, Remy-Jardin M, Myers JL et al (2018) Diagnosis of idiopathic pulmonary fibrosis. An Official ATS/ERS/JRS/ALAT Clinical Practice Guideline. Am J Respir Crit Care Med 198:e44–e68. 10.1164/rccm.201807-1255ST 30168753 10.1164/rccm.201807-1255ST

[74] Walsh SLF, Wells AU, Desai SR et al (2016) Multicentre evaluation of multidisciplinary team meeting agreement on diagnosis in diffuse parenchymal lung disease: a case-cohort study. Lancet Respir Med 4:557–565. 10.1016/S2213-2600(16)30033-9 27180021 10.1016/S2213-2600(16)30033-9

[75] Carlier S, Nasser M, Fort E et al (2022) Role of the occupational disease consultant in the multidisciplinary discussion of interstitial lung diseases. Respir Res 23:332. 10.1186/s12931-022-02257-6 36482462 10.1186/s12931-022-02257-6 PMC9733286

[76] Cox CW, Chung JH, Ackman JB et al (2020) ACR Appropriateness Criteria® Occupational Lung Diseases. J Am Coll Radiol 17:S188–S197. 10.1016/j.jacr.2020.01.022 32370962 10.1016/j.jacr.2020.01.022

[77] Tamur T, Suganuma N, Hering KG, Vehmas T, Itoh H, Akira M, Takashima Y, Hirano H, Kusaka Y (2015) Relationships (II) of international classification of high-resolution computed tomography for occupational and environmental respiratory diseases with ventilatory functions indices for parenchymal abnormalities. Ind Health 53(3):271–279. 10.2486/indhealth.2014-0072 10.2486/indhealth.2014-0072 PMC4466878 25810443

[78] Akgun M, Araz O, Akkurt I et al (2008) An epidemic of silicosis among former denim sandblasters. Eur Respir J 32:1295–1303. 10.1183/09031936.00093507 18579544 10.1183/09031936.00093507

[79] Chong S, Lee KS, Chung MJ et al (2006) Pneumoconiosis: comparison of imaging and pathologic findings. Radiogr a Rev Publ Radiol Soc North Am Inc 26:59–77. 10.1148/rg.261055070 10.1148/rg.261055070 16418244

[80] Sirajuddin A, Kanne JP (2009) Occupational lung disease. J Thorac Imaging 24:310–320. 10.1097/RTI.0b013e3181c1a9b3 19935227 10.1097/RTI.0b013e3181c1a9b3

[81] Koul A, Bawa RK, Kumar Y (2023) Artificial intelligence techniques to predict the airway disorders illness: a systematic review. Arch Comput Methods Eng State Art Rev 30:831–864. 10.1007/s11831-022-09818-4 10.1007/s11831-022-09818-4 PMC9516534 36189431

[82] Yang F, Tang Z-R, Chen J et al (2021) Pneumoconiosis computer aided diagnosis system based on X-rays and deep learning. BMC Med Imaging 21:189. 10.1186/s12880-021-00723-z 34879818 10.1186/s12880-021-00723-z PMC8653800

[83] Zhang L, Rong R, Li Q et al (2021) A deep learning-based model for screening and staging pneumoconiosis. Sci Rep 11:2201. 10.1038/s41598-020-77924-z 33500426 10.1038/s41598-020-77924-z PMC7838184

[84] Fazen LE, Linde B, Redlich CA (2020) Occupational lung diseases in the 21st century: the changing landscape and future challenges. Curr Opin Pulm Med 26:142–148. 10.1097/MCP.0000000000000658 31895883 10.1097/MCP.0000000000000658

[85] León-Jiménez A, Mánuel JM, García-Rojo M et al (2021) Compositional and structural analysis of engineered stones and inorganic particles in silicotic nodules of exposed workers. Part Fibre Toxicol 18:41. 10.1186/s12989-021-00434-x 34809667 10.1186/s12989-021-00434-x PMC8607701

[86] Guarnieri G, Bizzotto R, Gottardo O et al (2019) Multiorgan accelerated silicosis misdiagnosed as sarcoidosis in two workers exposed to quartz conglomerate dust. Occup Environ Med 76:178–180. 10.1136/oemed-2018-105462 30514749 10.1136/oemed-2018-105462

[87] Oliver LC, Zarnke AM (2021) Sarcoidosis: an occupational disease? Chest 160:1360–1367. 10.1016/j.chest.2021.06.003 34102140 10.1016/j.chest.2021.06.003 PMC8546237

[88] Schaad N, Berezowska S, Perren A, Hewer E (2024) Impact of template-based synoptic reporting on completeness of surgical pathology reports. Virchows Arch 484:31–36. 10.1007/s00428-023-03533-6 37017774 10.1007/s00428-023-03533-6 PMC10791929

[89] https://www.iccr-cancer.org/datasets/published-datasets/thorax/ website accessed 21.06.2023

[90] https://www.rcpath.org/profession/guidelines/cancer-datasets-and-tissue-pathways.html website accessed 21.06.2023

[91] Klebe S, Judge M, Brcic L et al (2024) Mesothelioma in the pleura, pericardium and peritoneum: recommendations from the International Collaboration on Cancer Reporting (ICCR). Histopathology 84:633–645. 10.1111/his.15106 38044849 10.1111/his.15106

[92] Banz Y, Berezowska S, de Leval L et al (2018) Advancing synoptic cancer reports beyond English: the University of Bern/PathoLink approach. Virchows Arch 473:655–656 30088082 10.1007/s00428-018-2431-0

